# Sentinel Enhanced Dengue Surveillance System — Puerto Rico, 2012–2022

**DOI:** 10.15585/mmwr.ss7303a1

**Published:** 2024-05-30

**Authors:** Zachary J. Madewell, Alfonso C. Hernandez-Romieu, Joshua M. Wong, Laura D. Zambrano, Hannah R. Volkman, Janice Perez-Padilla, Dania M. Rodriguez, Olga Lorenzi, Carla Espinet, Jorge Munoz-Jordan, Verónica M. Frasqueri-Quintana, Vanessa Rivera-Amill, Luisa I. Alvarado-Domenech, Diego Sainz, Jorge Bertran, Gabriela Paz-Bailey, Laura E. Adams

**Affiliations:** ^1^Division of Vector-Borne Diseases, CDC, San Juan, Puerto Rico; ^2^Ponce Health Sciences University, Ponce Research Institute, Ponce, Puerto Rico; ^3^Auxilio Mutuo Hospital, San Juan, Puerto Rico

## Abstract

**Problem/Condition:**

Dengue is the most prevalent mosquitoborne viral illness worldwide and is endemic in Puerto Rico. Dengue’s clinical spectrum can range from mild, undifferentiated febrile illness to hemorrhagic manifestations, shock, multiorgan failure, and death in severe cases. The disease presentation is nonspecific; therefore, various other illnesses (e.g., arboviral and respiratory pathogens) can cause similar clinical symptoms. Enhanced surveillance is necessary to determine disease prevalence, to characterize the epidemiology of severe disease, and to evaluate diagnostic and treatment practices to improve patient outcomes. The Sentinel Enhanced Dengue Surveillance System (SEDSS) was established to monitor trends of dengue and dengue-like acute febrile illnesses (AFIs), characterize the clinical course of disease, and serve as an early warning system for viral infections with epidemic potential.

**Reporting Period:**

May 2012–December 2022.

**Description of System:**

SEDSS conducts enhanced surveillance for dengue and other relevant AFIs in Puerto Rico. This report includes aggregated data collected from May 2012 through December 2022. SEDSS was launched in May 2012 with patients with AFIs from five health care facilities enrolled. The facilities included two emergency departments in tertiary acute care hospitals in the San Juan-Caguas-Guaynabo metropolitan area and Ponce, two secondary acute care hospitals in Carolina and Guayama, and one outpatient acute care clinic in Ponce. Patients arriving at any SEDSS site were eligible for enrollment if they reported having fever within the past 7 days. During the Zika epidemic (June 2016–June 2018), patients were eligible for enrollment if they had either rash and conjunctivitis, rash and arthralgia, or fever. Eligibility was expanded in April 2020 to include reported cough or shortness of breath within the past 14 days. Blood, urine, nasopharyngeal, and oropharyngeal specimens were collected at enrollment from all participants who consented. Diagnostic testing for dengue virus (DENV) serotypes 1–4, chikungunya virus, Zika virus, influenza A and B viruses, SARS-CoV-2, and five other respiratory viruses was performed by the CDC laboratory in San Juan.

**Results:**

During May 2012–December 2022, a total of 43,608 participants with diagnosed AFI were enrolled in SEDSS; a majority of participants (45.0%) were from Ponce. During the surveillance period, there were 1,432 confirmed or probable cases of dengue, 2,293 confirmed or probable cases of chikungunya, and 1,918 confirmed or probable cases of Zika. The epidemic curves of the three arboviruses indicate dengue is endemic; outbreaks of chikungunya and Zika were sporadic, with case counts peaking in late 2014 and 2016, respectively. The majority of commonly identified respiratory pathogens were influenza A virus (3,756), SARS-CoV-2 (1,586), human adenovirus (1,550), respiratory syncytial virus (1,489), influenza B virus (1,430), and human parainfluenza virus type 1 or 3 (1,401). A total of 5,502 participants had confirmed or probable arbovirus infection, 11,922 had confirmed respiratory virus infection, and 26,503 had AFI without any of the arboviruses or respiratory viruses examined.

**Interpretation:**

Dengue is endemic in Puerto Rico; however, incidence rates varied widely during the reporting period, with the last notable outbreak occurring during 2012–2013. DENV-1 was the predominant virus during the surveillance period; sporadic cases of DENV-4 also were reported. Puerto Rico experienced large outbreaks of chikungunya that peaked in 2014 and of Zika that peaked in 2016; few cases of both viruses have been reported since. Influenza A and respiratory syncytial virus seasonality patterns are distinct, with respiratory syncytial virus incidence typically reaching its annual peak a few weeks before influenza A. The emergence of SARS-CoV-2 led to a reduction in the circulation of other acute respiratory viruses.

**Public Health Action:**

SEDSS is the only site-based enhanced surveillance system designed to gather information on AFI cases in Puerto Rico. This report illustrates that SEDSS can be adapted to detect dengue, Zika, chikungunya, COVID-19, and influenza outbreaks, along with other seasonal acute respiratory viruses, underscoring the importance of recognizing signs and symptoms of relevant diseases and understanding transmission dynamics among these viruses. This report also describes fluctuations in disease incidence, highlighting the value of active surveillance, testing for a panel of acute respiratory viruses, and the importance of flexible and responsive surveillance systems in addressing evolving public health challenges. Various vector control strategies and vaccines are being considered or implemented in Puerto Rico, and data from ongoing trials and SEDSS might be integrated to better understand epidemiologic factors underlying transmission and risk mitigation approaches. Data from SEDSS might guide sampling strategies and implementation of future trials to prevent arbovirus transmission, particularly during the expansion of SEDSS throughout the island to improve geographic representation.

## Introduction

Dengue is an acute febrile viral illness transmitted by *Aedes* spp. mosquitoes and caused by four closely related dengue virus serotypes (DENV 1–4). DENV causes an estimated 390 million infections (*1*) and 40,500 deaths worldwide each year (*2*). In contrast to data suggesting that the prevalence of most communicable diseases is declining worldwide, dengue incidence and disease prevalence have been increasing over time, measured both by disability-adjusted life years and mortality attributed to dengue (*2*–*4*). The increased prevalence is most pronounced in Southeast Asia; dengue remains endemic in Puerto Rico, where large outbreaks typically occur in cycles of 3–7 years (*5*). The most recent epidemic in Puerto Rico occurred during 2012–2013, dominated by DENV-1; sporadic cases of DENV-4 also occurred. During 2010–2020, dengue was associated with approximately 30,000 confirmed and probable cases, including 584 severe cases, approximately 10,000 hospitalizations, and 68 deaths islandwide (*6*). The southern region of Puerto Rico, which includes Ponce, has historically had a higher incidence of dengue. Ponce is the fourth largest municipality on the island and experienced a substantial proportion of dengue cases during the 2012–2013 epidemic (*7*).

Suspected dengue cases have historically been reported to the Passive Dengue Surveillance System (PDSS), a system jointly managed by CDC and the Puerto Rico Department of Health (PRDH). These cases were reported based on clinical suspicion, followed by laboratory confirmation by PRDH. PDSS faces certain challenges (e.g., underreporting, limited testing of suspected cases, and incomplete clinical data), highlighting the need for complementary and timely surveillance methods. Dengue presents diagnostic challenges because its nonspecific symptoms overlap with those of other acute febrile illnesses (AFIs), emphasizing the need for reliable diagnostic tests. Surveillance systems are often limited by underdiagnosis and underreporting, thereby missing cases. In 2012, CDC transferred the operational responsibility of PDSS to PRDH and established the Sentinel Enhanced Dengue Surveillance System (SEDSS), a site-based active surveillance system used to monitor acute febrile and respiratory illnesses within certain emergency departments (EDs) (*8*–*10*). SEDSS was designed to diagnose and characterize dengue infections, along with arboviral and other respiratory viruses, accompanied by comprehensive demographic, syndromic, and clinical data. SEDSS addresses these challenges through enhanced surveillance and by providing comprehensive diagnostic testing for all patients with AFIs, allowing for capture of the full spectrum of case and disease prevalence. Since May 2012, SEDSS has demonstrated its adaptability, expanding its scope to cover emerging pathogens such as chikungunya virus (CHIKV) in 2014, Zika virus (ZIKV) in 2016, and SARS-CoV-2 in 2020. This real-time tracking capability has positioned SEDSS as a flexible and responsive sentinel system, and this report represents the first comprehensive summary of SEDSS data, marking a milestone in the reporting of these surveillance findings.

This report summarizes arboviral and respiratory illnesses among patients with AFIs seeking care at SEDSS sites from May 5, 2012, through December 31, 2022, and describes large outbreaks of dengue, chikungunya, Zika, and COVID-19 that occurred at different points during the surveillance period. This report also characterizes the relative prevalence of dengue warning signs and severe dengue in different age groups among laboratory-confirmed cases.

## Methods

### Surveillance Sites and Procedures

Data for this enhanced surveillance study were collected from patients with AFIs from hospitals participating in SEDSS from 2012 to 2022. Data collection adhered to a protocol approved by institutional review boards at CDC, Auxilio Mutuo, and Ponce Medical School Foundation with informed consent obtained from all participants. Recruitment for SEDSS began in May 2012 with two tertiary care facilities, two secondary care facilities, and one outpatient acute care clinic. During 2012–2015, SEDSS included three sites: 1) Saint Luke’s Episcopal Hospital (SLEH) in Ponce (SLEH-Ponce), a tertiary acute care hospital with 425 beds, an average of 50,000 patient visits per year, and 11,000 overall inpatient admissions per year; 2) SLEH-Guayama, a secondary acute care hospital with 116 beds, 40,000 patient visits per year, and 6,000 overall inpatient admissions per year; and 3) Hospital de La Universidad de Puerto Rico (UPR) in Carolina (UPR-Carolina), a secondary acute care teaching hospital with 250 beds, 55,000 medical visits per year, and 10,000 overall inpatient admissions per year (*9*–*11*). SLEH-Guayama participated from February 2013 to September 2015, UPR-Carolina operated from July 2013 to August 2015, and SLEH-Ponce continued participation through 2022. In 2016, Centro de Emergencia y Medicina Integrada (CEMI), an outpatient acute care clinic in Ponce registering approximately 1,700 annual clinic visits and 3,000 admissions joined the network. In November 2018, Auxilio Mutuo Hospital, a tertiary care facility in the San Juan-Caguas-Guaynabo metropolitan area (San Juan metro area) with 497 beds, 33,000 patient visits per year, and 8,000 overall inpatient admissions per year, was incorporated. The expansion to the San Juan metro area enhanced geographic representativeness, allowing the surveillance system to cover a broader and more diverse population within Puerto Rico.

Informational posters were available in hospital waiting rooms and hallways to inform the community about SEDSS. Potential participants were identified by triage nurses as any patient with an AFI defined by the presence of fever ≥100.4°F (≥38.0°C) for temperatures measured orally, ≥99.5°F (≥37.5°C) for temperatures measured rectally, and ≥101.3°F (≥38.5°C) for temperatures measured axillary at the time of triage or chief complaint of having a fever within the past 7 days. During the Zika epidemic (June 2016–June 2018), patients were eligible for enrollment in SEDSS if they visited a SEDSS site with either rash and conjunctivitis, rash and arthralgia, or fever (*12*). These criteria were applicable specifically during the Zika epidemic period to enhance Zika surveillance and are no longer part of the current eligibility criteria. Beginning in April 2020, patients reporting cough or dyspnea within the past 14 days (with or without fever) also were eligible for enrollment to better capture respiratory viruses (*9*). No age groups were excluded, although infants were only eligible for enrollment if they visited the hospital after their initial discharge after birth. All patients meeting the inclusion criteria were invited to participate in the study using convenience sampling. If a patient was incapacitated at the time of triage because of acute illness, the patient was only asked to participate after stabilization. All participants received an informational sheet about SEDSS and were enrolled after providing informed consent.

### Data Collection

Data from SEDSS were collected through patient interview and medical record review at intake during patient enrollment on a case investigation form (CIF) and approximately 7–14 days later on a convalescent sample processing form (CSPF). The CIF included demographic information (e.g., name, age, sex, pregnancy status, and contact information), comorbidities (e.g., diabetes, hypertension, asthma, chronic kidney disease, and immunodeficiency), and clinical features (e.g., onset date, vital signs, signs, and symptoms). The CSPF contained comparable information to the CIF but added the date of the second specimen collection and indicators of AFI severity (e.g., hospitalizations and other clinic visits). Data were originally collected using paper copies of the CIF and CSPF and were transitioned to electronic data in 2020 using the Research Electronic Data Capture (REDCap) system (REDCap Consortium; version 5.20.11) (*13*,*14*).

Inpatient medical data for participants with AFIs who were admitted to the hospital from SLEH-Ponce, SLEH-Guayama, and Auxilio Mutuo Hospital also were collected using a separate form (Hospital Admitted Abstraction Form) and entered into REDCap to collect key clinical indicators of disease severity and progression. For admitted patients, these data included information on extent and nature of hemorrhage, plasma leakage (e.g., ascites and pleural and cardiac effusions), hematologic indicators of increased intravascular permeability (e.g., hematocrit and serum albumin levels), additional blood pressure and heart rate measures to assess shock, and indicators of severe organ involvement (e.g., liver impairment, meningitis, and encephalitis) (*15*). These data were subsequently merged with data entered from the SEDSS CIF and CSPF forms using medical record numbers.

### Sample Collection, Laboratory Procedures, and Case Confirmation

Blood, nasopharyngeal (NP), and oropharyngeal (OP) specimens were collected at enrollment from eligible participants. Additional blood samples (e.g., serum and whole blood) also were collected during the convalescent phase. Participants were required to provide at least one sample (blood, OP, or NP) to participate. All participants had molecular testing for dengue, chikungunya (December 2013–April 2020), and Zika (December 2015–April 2020) viruses for specimens collected within 7 days of symptom onset. Before March 2016, DENV was detected by the CDC DENV-1–4 Real-Time Reverse Transcription-Polymerase Chain Reaction (RT-PCR) Assay (*16*). During March 2016–April 2020, the CDC Trioplex Real-Time RT-PCR Assay was used to detect DENV, CHIKV, and ZIKV concurrently, followed by the CDC DENV-1–4 Real-Time RT-PCR Assay on any samples positive for DENV to determine serotype (*17*). After April 2020, the CDC DENV-1–4 Real Time RT-PCR Assay again was used as the primary assay to detect DENV. Serologic testing was done by immunoglobulin M (IgM) antibody capture enzyme-linked immunosorbent assay for anti-DENV, anti-CHIKV (December 2013–March 2019), and anti-ZIKV (December 2015–March 2019) antibodies for specimens collected >3 days after symptom onset (*18*). Molecular diagnostic testing of NP and OP specimens was done by RT-PCR on a panel of respiratory viruses including influenza A (IAV) and influenza B (IBV) viruses, human adenovirus (HAdV), respiratory syncytial virus (RSV), human metapneumovirus (HMPV), human parainfluenza virus (HPIV) types 1 and 3, seasonal human coronavirus (HCoV [229E, OC43, NL63, and HKU1]) (*18*,*19*), and for SARS-CoV-2. For SARS-CoV-2, the RT-PCR assays used included the CDC Real-Time RT-PCR Panel for tests performed before December 2021 and the CDC Influenza SARS-CoV-2 Multiplex Assay for tests performed in December 2021 and later (*20*,*21*). HCoV testing was conducted consistently from 2012 to 2014 but was discontinued because of the viruses’ low frequency. However, sporadic testing based on clinical suspicion or exposure to known cases might have occurred throughout the study period. Molecular and serologic results were used to classify participants as having confirmed or probable CHIKV, DENV, or ZIKV infection using established guidance (*22*,*23*) (Table 1).

**TABLE 1:** Laboratory criteria for diagnosis of confirmed and probable dengue, chikungunya, and Zika virus infections, Puerto Rico, 2012–2022*

Classification	Confirmed or probable	Period	Nucleic acid amplification tests (reverse transcription polymerase chain reaction)	Immunoglobulin M enzyme-linked immunosorbent assay
DENV infection	Confirmed	2012–2022	DENV assay positive	DENV assay positive, negative, or not performed
	Probable	2012–2015	DENV assay negative or not performed	DENV assay positive
	Probable	2016–2022	DENV assay negative or not performed	DENV assay positive and ZIKV assay negative or not performed
CHIKV infection	Confirmed	2014–2022	CHIKV assay positive	CHIKV assay positive, negative, or not performed
	Probable	2014–2022	CHIKV assay negative or not performed	CHIKV assay positive
ZIKV infection	Confirmed	2016–2022	ZIKV assay positive	ZIKV assay positive, negative, or not performed
	Probable	2016–2022	ZIKV assay negative or not performed	ZIKV assay positive and DENV assay negative
Recent *flavivirus* infection	Probable	2016–2022	DENV assay negative or not performed and ZIKV assay negative or not performed	DENV assay positive and ZIKV assay positive, or DENV assay positive and ZIKV assay not performed, or DENV assay not performed and ZIKV assay positive
Arboviral co-infection	Confirmed	2012–2022	Viral assays positive for any two of the arboviruses tested Viral assays positive for all three of the arboviruses tested	Any combination of viral assays positive, negative, or not performed for the arboviruses tested

**Abbreviations:** DENV = dengue virus; CHIKV = chikungunya virus; ZIKV = Zika virus.

* **Sources:** Sharp TM, Fischer M, Muñoz-Jordán JL, et al. Dengue and Zika virus diagnostic testing for patients with a clinically compatible illness and risk for infection with both viruses. MMWR Recomm Rep. 2019 Jun 14;68:1-10. CDC. Arboviral diseases, neuroinvasive and non-neuroinvasive 2015 case definition [Internet]. Atlanta, GA: US Department of Health and Human Services, CDC; 2021. https://ndc.services.cdc.gov/case-definitions/arboviral-diseases-neuroinvasive-and-non-neuroinvasive-2015

Dengue warning signs and severe dengue were defined by the Pan American Health Organization (*24*), incorporating available clinical indicators from SEDSS intake and follow-up forms and abstracted inpatient medical records. Dengue warning signs were defined by abdominal pain or tenderness, persistent vomiting, pleural or pericardial effusion or ascites, mucosal bleeding, restlessness, or hepatomegaly (*15*). Severe dengue was defined as severe plasma leakage or shock, severe bleeding, or severe organ impairment (*15*).

### Analysis

Descriptive frequencies of AFI cases were reported by selected characteristics, including year, days post onset, age group, sex, ED encounter, physician diagnosis, clinical disposition, and chronic conditions. These characteristics also were reported by arboviral infection status (confirmed and probable DENV, ZIKV, and CHIKV) and respiratory virus infection (confirmed IAV, IBV, RSV, HAdV, HPIV-1, HPIV-3, HMPV, HCoV, and SARS-CoV-2). Clinical characteristics were reported by age group for participants with confirmed or probable DENV, CHIKV, and ZIKV infections. A patient might have been enrolled in SEDSS more than one time over the course of the study. The denominator for this analysis was the number of unique patient visits. Arbovirus and acute respiratory virus infection data were aggregated into weekly infection counts and plotted by epidemiologic week and year over the surveillance period. The frequency of symptoms for dengue, chikungunya, and Zika cases, as well as the frequency of warning signs and indicators of severe dengue, were calculated. The proportion of AFI cases positive for arboviruses or acute respiratory viruses by municipality is presented using choropleth maps with 5% equal intervals. All analyses were done using R software (R Foundation for Statistical Computing; version 4.3.1).

## Ethics Statement

The institutional review boards at CDC, Auxilio Mutuo, and Ponce Medical School Foundation approved the SEDSS study protocols 6,214 and 120308-VR, respectively. Written consent to participate was obtained from all adult participants and emancipated minors. For minors aged 14–20 years, written consent was obtained: for children aged 7–13 years, parental written consent and participant assent were obtained.

## Results

A total of 24,020 (27.2%) were recruited participants among 88,255 eligible patients from SLEH-Ponce (2012–2022), 2,893 (19.2%) among 15,071 from SLEH-Guayama (2013–2015), 9,240 (36.4%) recruited of 25,378 eligible from CEMI (2016–2022), 1,436 (23.6%) among 6,090 from UPR-Carolina (2013–2015), and 2,554 (10.6%) among 24,181 from Auxilio Mutuo Hospital (2021–2022) (Supplementary Table 2, https://stacks.cdc.gov/view/cdc/153721). The number of eligible patients was unavailable for Auxilio Mutuo Hospital (2018–2020); however, 2,733 participants were recruited during this period.

From May 2012 to December 2022, there were 43,608 unique visits from 35,855 total participants enrolled in SEDSS (Supplementary Figure 1, https://stacks.cdc.gov/view/cdc/153721), including 7,690 hospitalizations or transfers and 72 deaths (Table 2). Participants in SEDSS resided in 76 of the 78 municipalities in Puerto Rico; most visits were from participants residing in municipalities near the sentinel surveillance sites (e.g., Ponce [19,612 visits], Juana Díaz [3,535], Peñuelas [3,449], San Juan metro area [2,460], Villalba [1,719], Carolina [1,694], and Guayama [1,578]). Visits from participants were reported by residents of 18 U.S. states including Florida (nine), Texas (seven), and New York (six).

**TABLE 2:** Number and percentage of visits associated with acute febrile illness or suspected arboviral or acute respiratory viral illness, by selected characteristics — Sentinel Enhanced Dengue Surveillance System, Puerto Rico, 2012–2022

Characteristic	Male	Female	Total
	No. (%)	No. (%)	No. (%)
**Days post onset**			
0–3	16,276 (78.5)	17,421 (76.2)	**33,699 (77.3)**
4–7	2,706 (13.0)	3,382 (14.8)	**6,092 (14.0)**
>7	267 (1.3)	362 (1.6)	**629 (1.4)**
Unknown	1,491 (7.2)	1,696 (7.4)	**3,188 (7.3)**
**Age, yrs**			
<1	1,851 (8.9)	1,473 (6.4)	**3,324 (7.6)**
1–4	5,387 (26.0)	4,462 (19.5)	**9,849 (22.6)**
5–9	2,981 (14.4)	2,697 (11.8)	**5,679 (13.0)**
10–19	3,175 (15.3)	3,235 (14.2)	**6,411 (14.7)**
20–29	1,880 (9.1)	3,057 (13.4)	**4,938 (11.3)**
30–39	1,308 (6.3)	2,074 (9.1)	**3,383 (7.8)**
40–49	1,131 (5.5)	1,655 (7.2)	**2,786 (6.4)**
≥50	3,027 (14.6)	4,208 (18.4)	**7,238 (16.6)**
**Clinical disposition**			
Ambulatory	16,518 (79.6)	18,944 (82.9)	**35,467 (81.3)**
Admitted or transferred	4,014 (19.4)	3,675 (16.1)	**7,690 (17.6)**
Left against medical advice	74 (0.4)	96 (0.4)	**170 (0.4)**
Deceased	48 (0.2)	24 (0.1)	**72 (0.2)**
Unknown	86 (0.4)	122 (0.5)	**209 (0.5)**
**Co-existing medical condition**			
Diabetes	1547 (7.5)	1,991 (8.7)	**3,538 (8.1)**
Chronic pulmonary disease	3,919 (18.9)	4,561 (20.0)	**8,482 (19.5)**
Hypertension	2,478 (11.9)	3,294 (14.4)	**5,773 (13.2)**
Chronic kidney disease	286 (1.4)	262 (1.1)	**548 (1.3)**
Immunodeficiency	115 (0.6)	155 (0.7)	**270 (0.6)**
Sickle cell disease	53 (0.3)	139 (0.6)	**192 (0.4)**
Obesity**^†^**	3,072 (20.2)	4,547 (26.0)	**7,619 (23.3)**
**Total**	**20,740 (100.0)**	**22,861 (100.0)**	43,608* (100.0)

* Sex was not recorded for seven visits.

^†^ Among persons with available body mass index, n = 15,191 males, n = 17,501 females, and N = 32,698 total persons.

### Etiologic Agents

**FIGURE 1:**
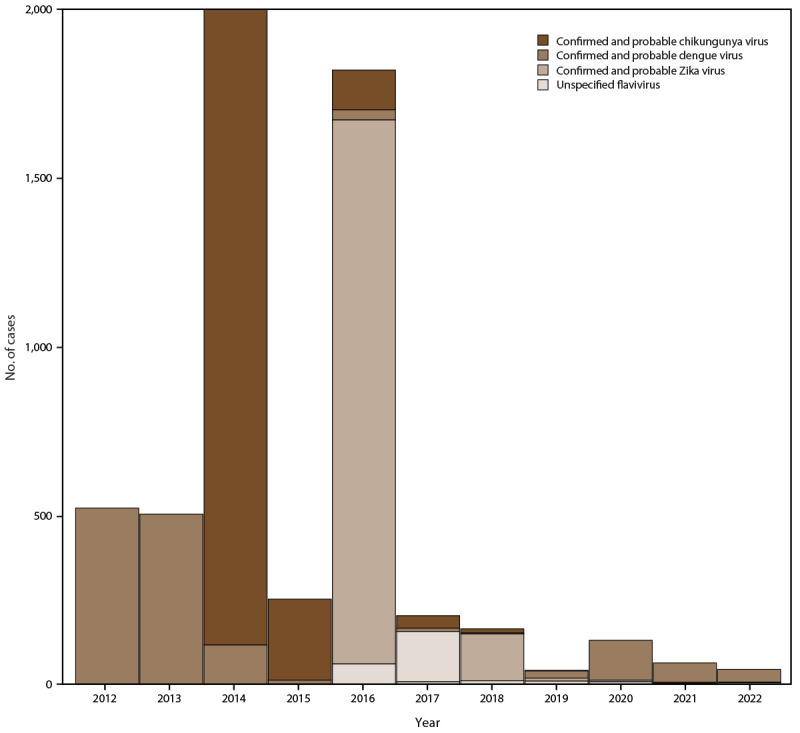
Confirmed or probable dengue, chikungunya, and Zika virus cases, by year — Sentinel Enhanced Dengue Surveillance System, Puerto Rico, May 2012–December 2022* * Cases were deemed to be laboratory-confirmed if a serum or urine specimen was polymerase chain reaction– or immunoglobulin M–positive for a particular arbovirus.

**FIGURE 2:**
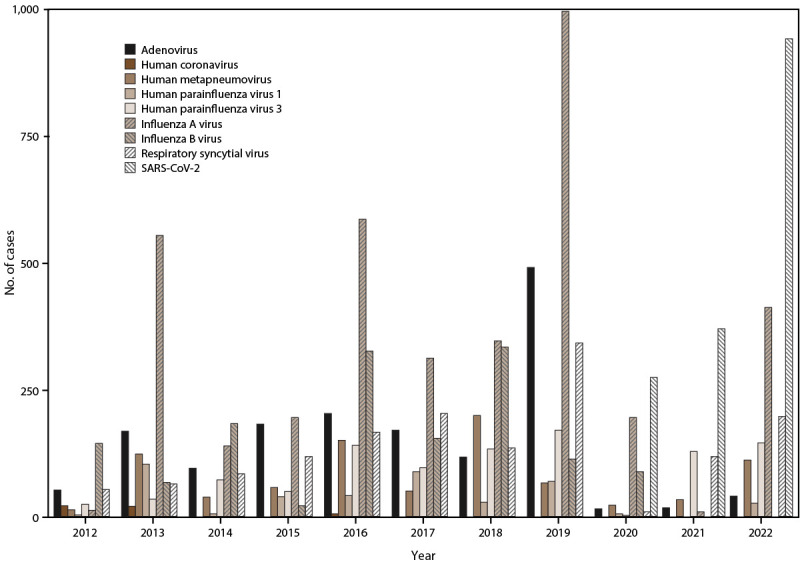
Acute respiratory virus cases, by year — Sentinel Enhanced Dengue Surveillance System, Puerto Rico, May 2012–December 2022* * Respiratory viruses were identified and confirmed by reverse transcription–polymerase chain reaction testing of nasopharyngeal swabs.

Laboratory testing identified positive results for arboviruses, including DENV (959 confirmed and 473 probable cases), ZIKV (1,449 confirmed and 469 probable cases), and CHIKV (1,804 confirmed and 489 probable cases) (Figure 1). In addition, positive results were identified for the following respiratory viruses: IAV (3,756 cases), IBV (1,430 cases), SARS-CoV-2 (1,586 cases), HAdV (1,550 cases), RSV (1,489 cases), HPIV-1 (407 cases), HPIV-3 (993 cases), HMPV (862 cases), and HCoV (46 cases) (Figure 2).

### Geographic Distribution and Temporal Trends

#### Arboviruses

**FIGURE 3:**
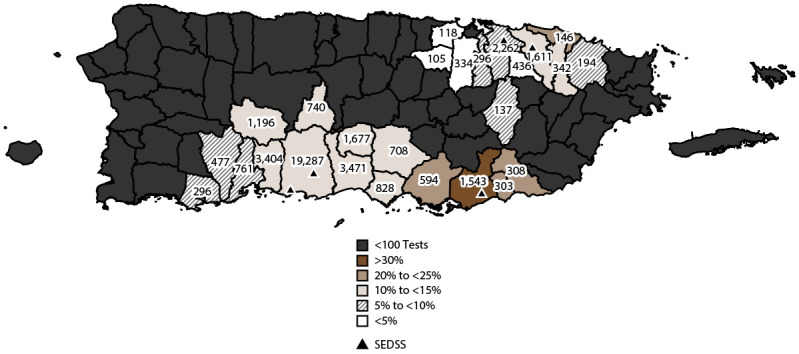
Number and proportion of acute febrile illness cases with participants testing positive for any arbovirus, by municipality — Sentinel Enhanced Dengue Surveillance System, Puerto Rico, 2012–2022*^,†^ **Abbreviation:** SEDSS = Sentinel Enhanced Dengue Surveillance System. * Arboviruses included dengue, chikungunya, and Zika viruses; the number of tests for any arbovirus in each municipality is shown. ^†^ The proportion of acute febrile illness cases testing positive for arboviruses is divided into intervals of equal size (5%).

The proportion of visits from participants testing positive for any arbovirus in municipalities with at least 100 participants tested was highest for those with residence in Guayama (539 of 1,543 [34.9%]), Salinas (144 of 594 [ 24.2%]), Patillas (64 of 308 [20.8%]), and Arroyo (62 of 303 [20.5%]) in southeast Puerto Rico and Loíza (33 of 146 [22.6%]) in northeast Puerto Rico (Figure 3). Conversely, municipalities that had the lowest proportion of visits from participants testing positive for arboviruses included Toa Baja (two of 118 [1.7%]) and Toa Alta (three of 105 [2.9%]) in northern Puerto Rico. The proportion of participants with AFI who tested positive for DENV were relatively consistent across PRDH regions (Supplementary Figure 2, https://stacks.cdc.gov/view/cdc/153721). The highest proportion of visits from participants testing positive for CHIKV was from San Juan metro area (296 of 2,481 [11.9%]) and Ponce (1,952 of 23,804 [8.2%]); the highest proportion who tested positive for ZIKV was from Ponce (1,886 of 19,324 [9.6%]).

**FIGURE 4:**
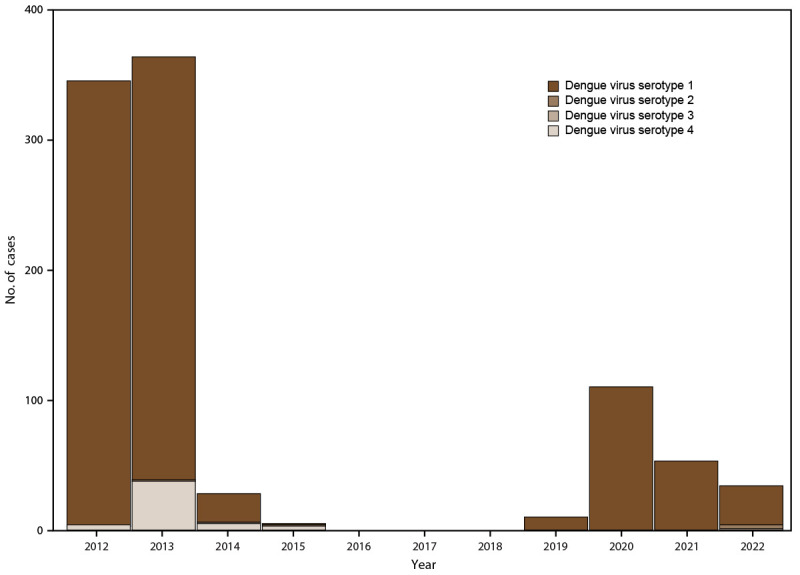
Confirmed dengue cases, by serotype and year — Sentinel Enhanced Dengue Surveillance System, Puerto Rico, May 2012 – December 2022* * Dengue cases were assigned a serotype if a participant’s serum specimen was polymerase chain reaction–positive for a specific dengue virus serotype.

Circulation of DENV, CHIKV, and ZIKV had minimal overlap, with transmission of each virus occurring during distinct periods (Supplementary Figure 3, https://stacks.cdc.gov/view/cdc/153721). SEDSS was initiated during an ongoing dengue outbreak in May 2012, and circulation continued until approximately February 2014. The majority of the 948 serotyped dengue cases were DENV-1 (891 [94.0%]), followed by DENV-4 (50 [5.3%]) (Figure 4) (Supplementary Figure 4, https://stacks.cdc.gov/view/cdc/153721). Six (0.6%) cases of DENV-2 and one (0.1%) case of DENV-3 were reported. CHIKV emerged on the island in January 2014 (*25*), and the first case was recorded in SEDSS in May 2014. Shortly after emergence, a large, time-limited outbreak of chikungunya occurred, which is reflected in the SEDSS data. During the peak of the chikungunya outbreak in September 2014, up to 195 cases per week were reported through SEDSS, representing 77.7% of all AFI cases in the system (Supplementary Figure 3, https://stacks.cdc.gov/view/cdc/153721). The outbreak eventually subsided in December 2014, with lingering low-level transmission occurring through November 2017. In December 2015, ZIKV emerged on the island, with the first case reported through SEDSS in January 2016. During the Zika outbreak peak, up to 101 cases per week were being reported through SEDSS before the outbreak ended in January 2017. In 2016, 1,612 of 6,301 (25.6%) of all ZIKV tests were positive (Supplementary Figure 5, https://stacks.cdc.gov/view/cdc/153721). ZIKV infections continued to be reported through SEDSS through 2018, albeit at low levels. During the Zika outbreak, 40 additional cases of probable flavivirus infections were reported, representing participants with cross-reactive IgM responses against ZIKV and DENV with no molecular evidence of infection. These cross-reactive IgM responses likely were associated with ZIKV because of the epidemiological context of a Zika outbreak and low DENV transmission during the same period. After the Zika outbreak subsided, low-to-no transmission of either ZIKV or DENV was observed until September 2019. Between September 2019 and December 2022, consistent low levels of weekly dengue cases (primarily DENV-1) were reported (Supplementary Figures 3 and 4, https://stacks.cdc.gov/view/cdc/153721), reaching a high of 23 cases observed in October 2020. Zero confirmed and nine probable ZIKV cases were reported from 2020 to 2022.

#### Respiratory Viruses

**FIGURE 5:**
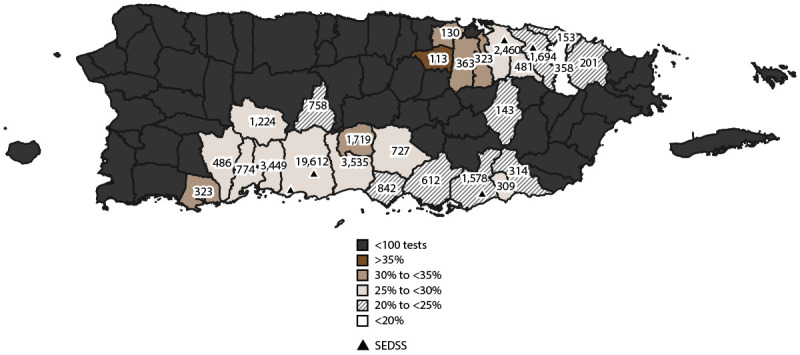
Number and proportion of acute febrile illness cases with participants testing positive for any acute respiratory virus, by municipality — Sentinel Enhanced Dengue Surveillance System, Puerto Rico, 2012–2022*^,†^ **Abbreviation:** SEDSS = Sentinel Enhanced Dengue Surveillance System. * Respiratory viruses included influenza A and B, respiratory syncytial virus, human adenovirus, human metapneumovirus, human parainfluenza viruses 1 and 3, human coronavirus, and SARS-CoV-2; the number of tests for any arbovirus in each municipality is shown. ^†^ The proportion of acute febrile illness cases testing positive for respiratory viruses is divided into intervals of equal size (5%).

The proportion of visits from participants testing positive for any acute respiratory virus in municipalities with at least 100 participants tested was highest for those with residence in Toa Alta (44 of 113;[38.9%]), Bayamón (135 of 363; [34.4%]), Guaynabo (102 of 323; [31.6%]), and Toa Baja (41 of 130; [31.5%]) in northern Puerto Rico, whereas Canóvanas (71 of 358; [19.8%]), Loíza (31 of 153; [20.3%]), and Río Grande (41 of 201; [20.4%]) in northeast Puerto Rico had the lowest proportions of visits from participants testing positive for respiratory viruses (Figure 5). The proportions of visits from participants with AFIs who tested positive for HAdV, HMPV, IAV, IBV, RSV, and HPIV-3 were relatively consistent across PRDH regions (Supplementary Figure 2, https://stacks.cdc.gov/view/cdc/153721). Only participants from Ponce, San Juan metro area, and Bayamón tested positive for HPIV-1 (Supplementary Figure 2, https://stacks.cdc.gov/view/cdc/153721). Percentage positivity for SARS-CoV-2 ranged from 13.3% (825 of 6,211) in Ponce to 32.1% (35 of 109) in Fajardo.

Seasonality was observed for both IAV and IBV, with IAV circulation typically preceding IBV circulation each year (Supplementary Figure 6, https://stacks.cdc.gov/view/cdc/153721). Periods of sustained IAV transmission typically spanned late November to early March, whereas IBV transmission typically occurred from mid-April to mid-June. Two notable exceptions occurred during which sustained IAV transmission was observed from July through October 2013 and IBV transmission from October 2016 to January 2017. Strong seasonality also was observed for confirmed RSV infections. Each year, RSV outbreaks typically began between late October and early November, with seasonal peaks in late November and early December. Circulation of HAdV, HMPV, HPIV-1, and HPIV-3 occurred throughout the year. Each virus still exhibited seasonality, with more pronounced HAdV transmission in July, HPIV-1 in October, HPIV-3 in April and May, and HMPV in December and January. In March 2020, all acute respiratory viruses decreased after the emergence of SARS-CoV-2. From March 2020 to April 2021, approximately all identified respiratory viral infections were SARS-CoV-2 after which other respiratory viruses reemerged. The highest weekly number of SARS-CoV-2 infections occurred in January 2022, coinciding with the emergence of Omicron. In 2022, a total of 942 of 3,988 (23.6%) tests for SARS-CoV-2 were positive (Supplementary Figure 5, https://stacks.cdc.gov/view/cdc/153721).

### Patient Characteristics, Signs, and Symptoms

#### Dengue Patient Characteristics, Symptoms, and Severity

A total of 1,432 dengue infections were identified among 1,427 study participants, which includes five participants who had two dengue infections during the study period (2012–2022). Of these infections, 1,026 (71.6%) occurred in 2012 and 2013 (Figure 1) (Supplementary Table 2, https://stacks.cdc.gov/view/cdc/153721). The majority of participants with dengue (765 [53.4%]) visited SEDSS sites within 3 days of symptom onset (Table 3). Most participants (764 [53.4%]) were male; among 668 female participants, 239 (35.8%) were women of childbearing age (15–44 years) and 11 were pregnant. A total of 587 (41.0%) participants with DENV infection were aged 10–19 years. Whereas 768 (53.6%) participants were ambulatory after the initial visit, 660 (46.1%) were admitted or transferred, and two participants died. The majority of common comorbidities were obesity (30.8%), hypertension (16.4%), chronic pulmonary disease (15.8%), and diabetes (9.6%). Before laboratory testing, 583 (40.7%) participants received a clinical diagnosis of dengue by a physician, 167 (11.7%) with a respiratory infection, and 560 (39.1%) with an unspecified infection. Among participants with DENV infection, 81 (5.7%) were coinfected with a respiratory virus, including 18 participants with HAdV and 28 with IAV or IBV.

**TABLE 3:** Number and percentage of cases of laboratory evidence of dengue, Zika, or chikungunya infection,* by selected characteristics —Sentinel Enhanced Dengue Surveillance System, Puerto Rico, 2012–2022

Characteristic	Dengue confirmed	Dengue probable	Dengue total	Chikungunya confirmed	Chikungunya probable	Chikungunya total	Zika confirmed	Zika probable	Zika total
	No. (%)	No. (%)	No. (%)	No. (%)	No. (%)	No. (%)	No. (%)	No. (%)	No. (%)
**Emergency department physician diagnosis**									
Dengue	441 (46.0)	142 (30.0)	**583 (40.7)**	142 (7.9)	32 (6.5)	**174 (7.6)**	16 (1.1)	5 (1.1)	**21 (1.1)**
Chikungunya	3 (0.3)	12 (2.5)	**15 (1.0)**	318 (17.6)	27 (5.5)	**345 (15.0)**	1 (0.1)	1 (0.2)	**2 (0.1)**
Zika	1 (0.1)	0 (—)	**1 (0.1)**	0 (—)	3 (0.6)	**3 (0.1)**	80 (5.5)	2 (0.4)	**82 (4.3)**
Influenza	32 (3.3)	24 (5.1)	**56 (3.9)**	165 (9.1)	53 (10.8)	**218 (9.5)**	6 (0.4)	60 (12.8)	**66 (3.4)**
Leptospirosis	9 (0.9)	2 (0.4)	**11 (0.8)**	6 (0.3)	4 (0.8)	**10 (0.4)**	3 (0.2)	10 (2.1)	**13 (0.7)**
Respiratory infections^†^	105 (10.9)	62 (13.1)	**167 (11.7)**	146 (8.1)	83 (17.0)	**229 (10.0)**	45 (3.1)	86 (18.3)	**131 (6.8)**
Infection not otherwise specified^§^	353 (36.8)	207 (43.8)	**560 (39.1)**	1,056 (58.5)	241 (49.3)	**1,297 (56.6)**	1,003 (69.2)	228 (48.6)	**1,231 (64.2)**
**Days post-onset**									
0–3	563 (58.7)	202 (42.7)	**765 (53.4)**	1,693 (93.8)	261 (53.4)	**1,954 (85.2)**	1,195 (82.5)	272 (58.0)	**1,467 (76.5)**
4–7	392 (40.9)	250 (52.9)	**642 (44.8)**	109 (6.0)	218 (44.6)	**327 (14.3)**	253 (17.5)	192 (40.9)	**445 (23.2)**
>7	4 (0.4)	21 (4.4)	**25 (1.7)**	2 (0.1)	10 (2.0)	**12 (0.5)**	1 (0.1)	5 (1.1)	**6 (0.3)**
**Sex**									
Male	522 (54.4)	242 (51.2)	**764 (53.4)**	857 (47.5)	238 (48.7)	**1,095 (47.8)**	608 (42.0)	203 (43.3)	**811 (42.3)**
Female	437 (45.6)	231 (48.8)	**668 (46.6)**	947 (52.5)	251 (51.3)	**1,198 (52.2)**	841 (58.0)	266 (56.7)	**1,107 (57.7)**
**Age, yrs**									
<1	16 (1.7)	12 (2.5)	**28 (2.0)**	80 (4.4)	27 (5.5)	**107 (4.7)**	26 (1.8)	6 (1.3)	**32 (1.7)**
1–4	63 (6.6)	36 (7.6)	**99 (6.9)**	214 (11.9)	95 (19.4)	**309 (13.5)**	77 (5.3)	23 (4.9)	**100 (5.2)**
5–9	148 (15.4)	67 (14.2)	**215 (15.0)**	202 (11.2)	44 (9.0)	**246 (10.7)**	100 (6.9)	54 (11.5)	**154 (8.0)**
10–19	427 (44.5)	160 (33.8)	**587 (41.0)**	347 (19.2)	79 (16.2)	**426 (18.6)**	242 (16.7)	95 (20.3)	**337 (17.6)**
20–29	120 (12.5)	49 (10.4)	**169 (11.8)**	231 (12.8)	50 (10.2)	**281 (12.3)**	307 (21.2)	63 (13.4)	**370 (19.3)**
30–39	60 (6.3)	26 (5.5)	**86 (6.0)**	155 (8.6)	38 (7.8)	**193 (8.4)**	217 (15.0)	44 (9.4)	**261 (13.6)**
40–49	46 (4.8)	25 (5.3)	**71 (5.0)**	148 (8.2)	38 (7.8)	**186 (8.1)**	162 (11.2)	46 (9.8)	**208 (10.8)**
≥50	79 (8.2)	98 (20.7)	**177 (12.4)**	427 (23.7)	118 (24.1)	**545 (23.8)**	318 (21.9)	138 (29.4)	**456 (23.8)**
**Clinical disposition**									
Ambulatory	499 (52.0)	269 (56.9)	**768 (53.6)**	1,554 (86.1)	369 (75.5)	**1,923 (83.9)**	1,377 (95.0)	393 (83.8)	**1,770 (92.3)**
Admitted or transferred	459 (47.9)	201 (42.5)	**660 (46.1)**	237 (13.1)	100 (20.4)	**337 (14.7)**	51 (3.5)	66 (14.1)	**117 (6.1)**
Left against medical advice	0 (—)	0 (—)	**0 (—)**	0 (—)	1 ( 0.2)	**1 ( 0.0)**	20 (1.4)	5 (1.1)	**25 (1.3)**
Deceased	1 (0.1)	1 (0.2)	**2 (0.1)**	2 (0.1)	1 (0.2)	**3 (0.1)**	0 (—)	1 (0.2)	**1 (0.1)**
Unknown	0 (—)	2 (0.4)	**2 (0.1)**	11 (0.6)	18 (3.7)	**29 (1.3)**	1 (0.1)	4 (0.9)	**5 (0.3)**
**Chronic conditions**									
Diabetes	44 (4.6)	53 (11.2)	**97 (6.8)**	174 (9.6)	52 (10.6)	**226 (9.9)**	156 (10.8)	68 (14.5)	**224 (11.7)**
Chronic pulmonary disease	179 (18.7)	108 (22.8)	**287 (20.0)**	285 (15.8)	74 (15.1)	**359 (15.7)**	237 (16.4)	108 (23.0)	**345 (18.0)**
Hypertension	63 (6.6)	73 (15.4)	**136 (9.5)**	296 (16.4)	81 (16.6)	**377 (16.4)**	248 (17.1)	126 (26.9)	**374 (19.5)**
Chronic kidney disease	5 (0.5)	6 (1.3)	**11 (0.8)**	21 (1.2)	9 (1.8)	**30 (1.3)**	10 (0.7)	3 (0.6)	**13 (0.7)**
Immunodeficiency	7 (0.7)	5 (1.1)	**12 (0.8)**	12 (0.7)	4 (0.8)	**16 (0.7)**	12 (0.8)	4 (0.9)	**16 (0.8)**
Sickle cell disease	4 (0.4)	3 (0.6)	**7 (0.5)**	11 (0.6)	2 (0.4)	**13 (0.6)**	3 (0.2)	3 (0.6)	**6 (0.3)**
Obesity^¶^	97 (26.4)	68 (31.9)	**165 (28.4)**	199 (30.8)	86 (17.6)	**285 (29.6)**	466 (32.5)	128 (27.9)	**594 (31.4)**
Pregnant**									
Yes	5 (2.9)	6 (9.4)	**11 (4.6)**	55 (15.8)	8 (7.9)	**63 (14.0)**	67 (15.8)	10 (8.3)	**77 (14.2)**
No	143 (81.7)	52 (81.2)	**195 (81.6)**	260 (74.5)	85 (84.2)	**345 (76.7)**	351 (82.8)	107 (89.2)	**458 (84.2)**
Unknown	27 (15.4)	6 (9.4)	**33 (13.8)**	34 (9.7)	8 (7.9)	**42 (9.3)**	6 (1.4)	3 (2.5)	**9 (1.6)**
Visits from women aged 15–44 years, no.	175	64	**239**	349	101	**450**	424	120	**544**
**Coinfections**									
Arboviral co-infection	0 (—)	55 (11.6)	**55 (3.8)**	1 (0.1)	43 (8.8)	**44 (1.9)**	1 (0.1)	3 (0.6)	**4 (0.2)**
Respiratory virus co-infection^††^	34 (3.5)	47 (9.9)	**81 (5.7)**	34 (1.9)	57 (11.7)	**91 (4.0)**	41 (2.8)	111 (23.7)	**152 (7.9)**
**Total**	**959 (100.0)**	**473 (100.0)**	**1,432 (100.0)**	**1,804 (100.0)**	**489 (100.0)**	**2,293 (100.0)**	**1,449 (100.0)**	**469 (100.0)**	**1,918 (100.0)**

**Abbreviations:** DENV = dengue virus; IgM = immunoglobulin M; ZIKV = Zika virus.

* Confirmed infection for the three arboviruses was by positive reverse transcription-polymerase chain reaction results. Before Zika, probable dengue included positive IgM tests. After Zika emerged, probable dengue included IgM positivity for DENV along with negative IgM results for ZIKV because of notable cross-reactivity between dengue IgM and Zika IgM antibodies in serologic tests. Probable Zika included IgM positivity for ZIKV along with negative IgM results for DENV. Excludes unspecified flavivirus infections.

^†^ Respiratory infections include pneumonia, upper respiratory tract infections, bronchitis, bronchiolitis, respiratory syncytial virus, and COVID-19.

^§^ Infections not otherwise specified include urinary tract infections, gastroenteritis, pharyngitis, fever unspecified, viral syndrome, tonsillitis, otitis media, sinusitis, impetigo, cellulitis, infected wound, lymphadenopathy cervical, herpangina, febrile seizure, meningitis, sepsis, bacteremia, encephalitis, pancreatitis, myositis, conjunctivitis, myocarditis, Guillain-Barré syndrome, mononucleosis, and pyelonephritis.

^¶^ Among persons with available body mass index: dengue (n = 581), Zika (n = 1,893), and chikungunya (n = 962).

** Denominators for these analyses include visits from women of childbearing age defined as any female aged 15–44 years.

^††^ Respiratory virus coinfections included adenovirus, human parainfluenza virus (types 1 and 3), influenza virus (types A and B), respiratory syncytial virus (types A and B), human metapneumovirus, human coronavirus (HcoV-229E, HcoV-HKU1, HcoV-NL63, and HcoV-OC43), and SARS-CoV-2.

**FIGURE 6:**
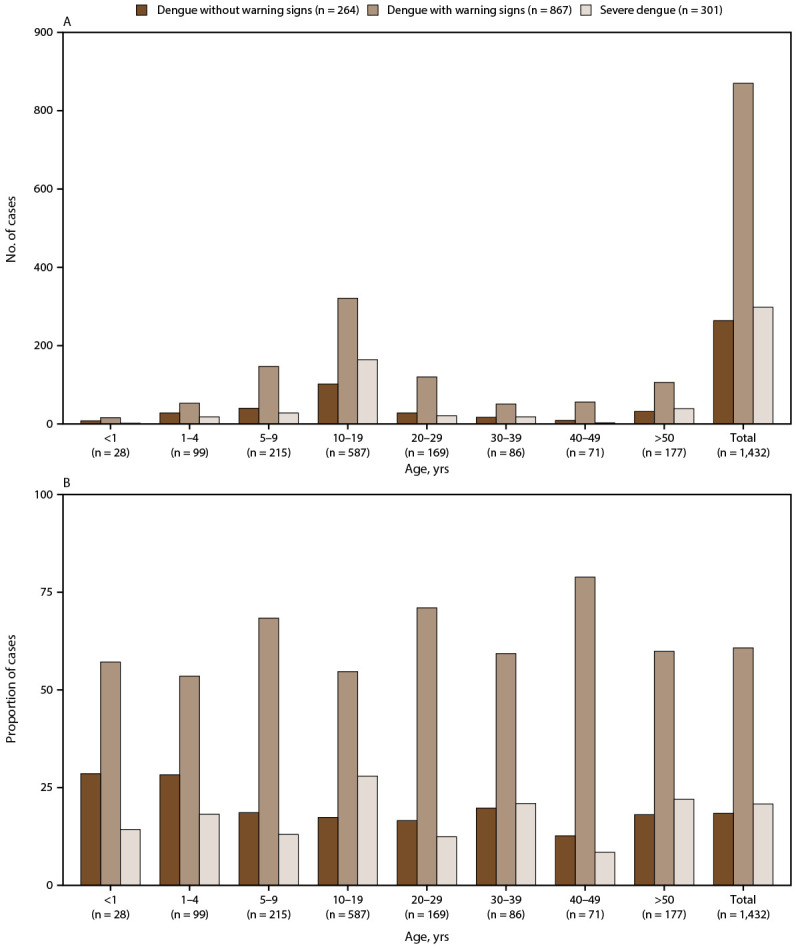
Number and proportion of dengue cases, by participant age group without warning signs, with warning signs without progressing to severe dengue, and severe dengue — Sentinel Enhanced Dengue Surveillance System, Puerto Rico, 2012–2022

Among the 1,432 participants with dengue reported through SEDSS, 263 (18.4%) received a diagnosis of dengue without warning signs, 788 (55.0%) received a diagnosis of dengue with warning signs and did not progress to severe dengue, and 381 (26.6%) received a diagnosis of severe dengue; two participants (0.1%) died. Among the 263 participants without warning signs, 788 with any warning sign, and 381 with severe dengue, 48 (18.3%), 293 (37.2%), and 319 (83.7%) were hospitalized or transferred, respectively. The distribution of dengue without warning signs, dengue with warning signs, and severe dengue was consistent across age groups with the exception that severe dengue was less prevalent among infants (Figure 6). Among the 557 participants with warning signs or severe dengue who were not hospitalized, 98 (17.6%) received a correct initial physician diagnosis of dengue. Age distribution of 1) specific clinical indicators of severity and 2) dengue without warning signs, dengue with warning signs, and severe dengue are presented (Table 4) (Figure 6). The most frequently reported general symptoms of dengue, in addition to fever, were headache (1,200 [83.8%]), myalgia (1,066 [74.4%]), and leukopenia (1,022 [71.4%]); however, all general symptoms, including fever, chills, nausea, rash, headache, retro-orbital pain, myalgia, arthralgia, and leukopenia, were reported in more than half of all participants with laboratory evidence of infection (Supplementary Figure 7, https://stacks.cdc.gov/view/cdc/153721). The most reported warning signs were abdominal pain (869 [60.7%]) and restlessness (699 [48.8%]). The most reported manifestation of severe dengue was severe plasma leakage (252 [17.6%]). Shock occurred in 32 (2.2%) participants. Severe organ impairment occurred in 57 (4.0%) participants, including 17 (1.2%) with liver impairment, seven (0.5%) with encephalopathy, seven (0.5%) with hepatitis, and one (0.1%) with aseptic meningitis. Seizures occurred in 26 (1.8%) participants (Table 4). Among the 11 pregnant women with laboratory evidence of DENV infection reported through SEDSS, seven (63.6%) had warning signs with no progression to severe dengue, and four (36.4%) had signs of severe dengue. Nine (81.2%) pregnant women were hospitalized.

**TABLE 4:** Number and percentage of cases of confirmed or probable dengue and clinical characteristics, by patient age — Sentinel Enhanced Dengue Surveillance System, Puerto Rico, 2012–2022

Symptom	<1 yr	1–4 yrs	5–9 yrs	10–19 yrs	20–29 yrs	30–39 yrs	40–49 yrs	≥50 yrs	Total
	No. (%)	No. (%)	No. (%)	No. (%)	No. (%)	No. (%)	No. (%)	No. (%)	No. (%)
**General symptom**									
Fever*	28 (100.0)	99 (100.0)	215 (100.0)	586 (99.8)	169 (100.0)	86 (100.0)	71 (100.0)	171 (96.6)	**1,425 (99.5)**
Chills	12 (42.9)	39 (39.4)	154 (71.6)	497 (84.7)	151 (89.3)	77 (89.5)	64 (90.1)	148 (83.6)	**1,142 (79.7)**
Nausea	10 (35.7)	44 (44.4	139 (64.7)	438 (74.6)	133 (78.7)	65 (75.6)	52 (73.2)	103 (58.2)	**984 (68.7)**
Rash	14 (50.0)	51 (51.5)	138 (64.2)	372 (63.4)	97 (57.4)	45 (52.3)	34 (47.9)	50 (28.2)	**801 (55.9)**
Headache	5 (17.9)	37 (37.4)	176 (81.9)	541 (92.2)	155 (91.7)	83 (96.5)	67 (94.4)	136 (76.8)	**1,200 (83.8)**
Retro-orbital pain	5 (17.9)	16 (16.2)	98 (45.6)	398 (67.8)	129 (76.3)	74 (86.0)	52 (73.2)	97 (54.8)	**869 (60.7)**
Myalgia	10 (35.7)	31 (31.3)	133 (61.9)	448 (76.3)	147 (87.0)	79 (91.9)	68 (95.8)	150 (84.7)	**1,066 (74.4)**
Arthralgia	4 (14.3)	20 (20.2)	112 (52.1)	351 (59.8)	136 (80.5)	73 (84.9)	60 (84.5)	141 (79.7)	**897 (62.6)**
Tachypnea^†^	2 (7.1)	7 (7.1)	57 (26.5)	336 (57.2)	72 (42.6)	48 (55.8)	27 (38.0)	76 (42.9)	**625 (43.6)**
Leukopenia (white blood cells <4,000/mm^3^) (N = 1,389)	3 (11.5)	36 (38.7)	135 (64.9)	431 (75.3)	109 (66.5)	53 (61.6)	39 (56.5)	63 (36.8)	**869 (62.6)**
**Warning sign**									
Persistent vomiting (three or more times in 24 hours)	4 (14.3)	22 (22.2)	59 (27.4)	167 (28.4)	59 (34.9)	19 (22.1)	15 (21.1)	32 (18.1)	**377 (26.3)**
Abdominal pain	5 (17.9)	41 (41.4)	143 (66.5)	389 (66.3)	110 (65.1)	49 (57.0)	41 (57.7)	91 (51.4)	**869 (60.7)**
Clinical fluid accumulation^§^	2 (7.1)	4 (4.0)	5 (2.3)	24 (4.1)	8 (4.7)	7 (8.1)	4 (5.6)	15 (8.5)	**69 (4.8)**
Mucosal bleeding^¶^	0 (—)	5 (5.1)	21 (9.8)	95 (16.2)	37 (21.9)	12 (14.0)	14 (19.7)	19 (10.7)	**203 (14.2)**
Restlessness**	14 (50.0)	42 (42.4)	92 (42.8)	263 (44.8)	88 (52.1)	47 (54.7)	42 (59.2)	111 (62.7)	**699 (48.8)**
**Indicator of severe dengue**									
Severe plasma leakage or shock	1 (3.6)	4 (4.0)	23 (10.7)	165 (28.1	16 (9.5)	16 (18.6)	5 (7.0)	34 (19.2)	**264 (18.4)**
Severe plasma leakage or intravascular permeability^††^	1 (3.6)	4 (4.0)	23 (10.7)	157 (26.7)	16 (9.5)	14 (16.3)	5 (7.0)	32 (18.1)	**252 (17.6)**
Ascites	1 (3.6)	0 (—)	1 (0.5)	2 (0.3)	1 (0.6)	1 (1.2)	0 (—)	2 (1.1)	**8 (0.6)**
Pleural or pericardial effusion	2 (7.1)	4 (4.0)	2 (0.9)	16 (2.7)	1 (0.6)	1 (1.2)	1 (1.4)	4 (2.3)	**32 (2.2)**
Hemoconcentration^§§^	5 (17.9)	5 (5.1)	6 (2.8)	44 (7.5)	17 (10.1)	9 (10.5)	11 (15.5)	27 (15.3)	**124 (8.7)**
Low albumin (N = 699)^¶¶^	2 (14.3)	22 (61.1)	63 (62.4)	218 (66.7)	32 (41.6)	19 (48.7)	14 (42.4)	44 (61.1)	**414 (59.2)**
Shock***	0 (—)	0 (—)	1 (0.5)	19 (3.2)	0 (—)	4 (4.7)	1 (1.4)	6 (3.4)	**31 (2.2)**
Hypotension (N = 1400)^†††^	0 (—)	1 (1.1)	20 (9.4)	77 (13.4)	10 (6.1)	4 (4.7)	4 (5.7)	8 (4.6)	**124 (8.9)**
Drop in systolic blood pressure >40 mmHg (N = 845)	2 (13.3)	0 (—)	4 (3.2)	13 (3.7)	2 (1.9)	5 (9.4)	2 (4.5)	16 (15.0)	**44 (5.2)**
Narrow pulse pressure	1 (3.6)	3 (3.0)	6 (2.8)	15 (2.6)	0 (—)	0 (—)	1 (1.4)	3 (1.7)	**29 (2.0)**
Capillary refill (>2 seconds)	0 (—)	4 (4.0)	10 (4.7)	42 (7.2)	14 (8.3)	5 (5.8)	5 (7.0)	14 (7.9)	**94 (6.6)**
Pale or cold skin	8 (28.6)	37 (37.4)	97 (45.1)	308 (52.5)	90 (53.3)	46 (53.5)	38 (53.5)	91 (51.4)	**715 (49.9)**
Blue skin or lips	0 (—)	2 (2.0)	7 (3.3)	40 (6.8)	7 (4.1)	5 (5.8)	1 (1.4)	2 (1.1)	**64 (4.5)**
Tachycardia^§§§^	2 (7.1)	24 (24.2)	29 (13.5)	139 (23.7)	67 (39.6)	29 (33.7)	29 (40.8)	40 (22.6)	**359 (25.1)**
Use of vasopressors, inotropic, vasodilators, or albumin	2 (7.1)	1 (1.0)	1 (0.5)	9 (1.5)	0 (—)	3 (3.5)	0 (—)	0 (—)	**16 (1.1)**
Hemorrhage	4 (14.3)	2 (2.0)	18 (8.4)	40 (6.8)	20 (11.8)	11 (12.8)	10 (14.1)	26 (14.7)	**131 (9.1)**
Gastrointestinal bleeding	3 (10.7)	1 (1.0)	13 (6.0)	25 (4.3)	10 (5.9)	5 (5.8)	7 (9.9)	19 (10.7)	**83 (5.8)**
Vomit with blood	0 (—)	1 (1.0)	6 (2.8)	14 (2.4)	10 (5.9)	4 (4.7)	1 (1.4)	3 (1.7)	**39 (2.7)**
Pulmonary hemorrhage	0 (—)	0 (—)	0 (—)	2 (0.3	0 (—)	0 (—)	0 (—)	0 (—)	**2 (0.1)**
Any blood product received	1 (3.6)	0 (—)	0 (—)	9 (1.5)	2 (1.2)	2 (2.3)	2 (2.8)	6 (3.4)	**22 (1.5)**
Severe organ involvement	1 (3.6)	4 (4.0)	5 (2.3)	30 (5.1)	4 (2.4)	2 (2.3)	3 (4.2)	8 (4.5)	**57 (4.0)**
Liver impairment (aspartate aminotransferase or alanine aminotransferase >1,000 IU/L) (N = 793)	0 (—)	0 (—)	3 (2.5)	11 (2.9)	2 (2.3)	0 (—)	1 (2.9)	0 (—)	**17 (2.1)**
Seizures	0 (—)	4 (4.0)	3 (1.4)	11 (1.9)	2 (1.2)	2 (2.3)	0 (—)	4 (2.3)	**26 (1.8)**
Intubation or mechanical ventilation	0 (—)	0 (—)	0 (—)	1 (0.2)	0 (—)	0 (—)	0 (—)	1 (0.6)	**2 (0.1)**
Prothrombin time and international normalized ratio ≥1.5 (N = 90)	0 (—)	0 (—)	1 (9.1)	0 (—)	0 (—)	0 (—)	2 (50.0)	2 (18.2)	**5 (5.6)**
Aseptic meningitis	0 (—)	0 (—)	0 (—)	1 (0.2)	0 (—)	0 (—)	0 (—)	0 (—)	**1 (0.1)**
Encephalopathy	0 (—)	1 (1.0)	0 (—)	4 (0.7)	0 (—)	0 (—)	0 (—)	2 (1.1)	**7 (0.5)**
**Total**	**28 (100.0)**	**99 (100.0)**	**215 (100.0)**	**587 (100.0)**	**169 (100.0)**	**86 (100.0)**	**71 (100.0)**	**177 (100.0)**	**1,432 (100.0)**

**Abbreviation:** bpm = beats per minute.

* Fever defined as either fever during the visit or in past 7 days

^†^ Tachypnea is an age-specific variable defined by respiratory rate (breaths per minute). Persons aged 0–3 months: >60 breaths per minute; persons aged 4–6 months: >45 breaths per minute; persons aged 7–12 months: >40 breaths per minute; persons aged 13 months–3 years: >30 breaths per minute; persons aged 4–6 years: >25 breaths per minute; persons aged 7–12 years: >22 breaths per minute; and persons aged >13 years: >18 breaths per minute.

^§^ Clinical fluid accumulation included pleural effusion, ascites, peritoneal fluid, pericardial effusion, free fluid, and swollen hands.

^¶^ Mucosal bleeding defined as bleeding from gums or nose, vaginal bleeding, or hematuria.

** Restlessness defined as nervousness, agitation, irritability, or disorientation.

^††^ Severe plasma leakage defined as respiratory distress and plasma leakage. Respiratory compromise defined as tachypnea, respiratory distress, accessory muscles use, supplemental oxygen, or intubation. Plasma leakage defined as pleural effusion, pericardial effusion, ascites, free fluid in abdomen, hematocrit change >20% during illness, hematocrit value >20% above baseline for age and sex at any time, or albumin low for age.

^§§^ Hemoconcentration defined as hematocrit change ≥20% during illness or hematocrit value ≥20% above baseline for age and sex at any time. Baseline values (females and males): 0–7 days: <61.2; 8–29 days: <52.8; 30–59 days: <42; 60 days – <3 years: <43.2; 3 – <6 years: <44; 6 – <9 years: <45.6; 9 – <12 years: <46.8; Males: 12 – <15 years: <50.4; 15 – <50 years: <54; 50 – <60 years: <52.8; ≥60 years: <51.6; Females: 12 – <15 years: <48; 15 – <50 years: 46.8; >50 years: <48.

^¶¶^ Low albumin defined based on age-specific criteria. If albumin level falls below the specified cut-off values (g/dL) by age: 0–15 days: <3.0; 15 days–1 year: <2.2; >1–15 years: <3.6; 16–18 years: <3.9; ≥19 years: <3.2.

*** Shock is defined as 1) shock listed in medical assessment at any time; 2) use of vasopressors, inotropes, or vasodilators, or albumin; 3) pulse pressure <20 mmHg or hypotension or drop in systolic blood pressure >40 mmHg and any two of the following: elevated heart rate, capillary refill >2 seconds, mottled skin, thready, weak pulse, pale or cold skin, blue skin or lips, cyanotic limbs, or cold limbs. **Source:** Paz-Bailey G, Sánchez-González L, Torres-Velasquez B, et al. Predominance of severe plasma leakage in pediatric patients with severe dengue in Puerto Rico. J Infect Dis 2022;226:1949–58.

^†††^ For persons aged ≥10 years, hypotension is defined as systolic blood pressure of <90 mmHg or mean arterial pressure <70 mmHg. Hypotension in children aged <10 years defined as systolic blood pressure of <70 + (age in years x 2) mmHg.

^§§§^ Tachycardia is an age-specific variable that might be an indicator of hypovolemia and dengue severity when combined with indicators of plasma leakage. A participant was defined as tachycardic at the following age-specific variables, according to *Nelson's Essentials of Pediatrics* (2002): <1 day: >154 bpm; 1–2 days: >159 bpm; 3–6 days: >166 bpm; 1–3 weeks: >182 bpm; 1–2 months: >179 bpm; 3–5 months: >186 bpm; 6–11 months: >169 bpm; 1–2 years: >151 bpm; 3–4 years: >137 bpm; 5–7 years: >133 bpm; 8–11 years: >130 bpm; 12–15 years: >119 years; >16 years: >100 bpm.

#### Chikungunya Patient Characteristics and Symptoms

Among 2,293 confirmed or probable chikungunya cases reported to SEDSS, 1,884 (82.2%) were reported in 2014 (Figure 1) (Supplementary Table 2, https://stacks.cdc.gov/view/cdc/153721). Most participants (1,954 [85.2%]) visited SEDSS sites within 3 days of symptom onset (Table 3). A majority of participants were female (1,198 [52.2%]), and among the 450 women of childbearing age, 63 (14.0%) were pregnant. The plurality of cases with laboratory evidence of CHIKV infection were among participants aged ≥50 years (545 [23.8%]), followed by those aged 10–19 years (426 [18.6%]), those aged 1–4 years (309 [13.5%]), and those aged 20–29 years (281 [12.3%]). Most participants (1,923 [83.9%]) were discharged after the initial visit, whereas 337 (14.7%) were either admitted or transferred; three (0.1%) participants died. The most common comorbidities were hypertension (377 [16.4%]), asthma (354 [15.4%]), and diabetes (226 [9.9%]). Before laboratory testing, 345 (15.0%) participants received a clinical diagnosis of chikungunya by a physician after triage, 229 (10.0%) with a respiratory infection, and 1,297 (56.6%) with an unspecified infection. Among participants with CHIKV infection, 91 (4.0%) were coinfected with a respiratory virus, including 44 with IAV or IBV.

In addition to fever, (2,271 [99.0%]) and chills (1,668 [72.7%]), the most reported symptoms among the 2,293 participants with laboratory evidence of CHIKV infection were myalgia (1,726 [76.5%]), arthralgia (1,726 [75.3%]), and headache (1,664 [72.6%]) (Table 5) (Supplementary Figure 7, https://stacks.cdc.gov/view/cdc/153721). The proportion of participants reporting headache, myalgia, and arthralgia consistently increased with age (p≤0.027); however, young children might be less likely to self-report certain of these symptoms.

**TABLE 5:** Number and percentage of cases of confirmed or probable chikungunya virus infection and clinical characteristics, by age — Sentinel Enhanced Dengue Surveillance System, Puerto Rico, 2012–2022

Symptom	<1 yr	1–4 yrs	5–9 yrs	10–19 yrs	20–29 yrs	30–39 yrs	40–49 yrs	≥50 yrs	Total
	No. (%)	No. (%)	No. (%)	No. (%)	No. (%)	No. (%)	No. (%)	No. (%)	No. (%)
Fever*	103 (96.3)	308 (99.7)	246 (100.0)	423 (99.3)	280 (99.6)	190 (98.4)	183 (98.4)	538 (98.7)	**2,271 (99.0)**
Chills	52 (48.6)	190 (61.5)	171 (69.5)	309 (72.5)	207 (73.7)	163 (84.5)	152 (81.7)	424 (77.8)	**1,668 (72.7)**
Nausea	26 (24.3)	129 (41.7)	111 (45.1)	234 (54.9)	139 (49.5)	114 (59.1)	107 (57.5)	269 (49.4)	**1,129 (49.2)**
Rash	75 (70.1)	185 (59.9)	163 (66.3)	305 (71.6)	195 (69.4)	134 (69.4)	104 (55.9)	257 (47.2)	**1,418 (61.8)**
Headache	9 (8.4)	112 (36.2)	168 (68.3)	365 (85.7)	249 (88.6)	173 (89.6)	167 (89.8)	421 (77.2)	**1,664 (72.6)**
Eye pain	9 (8.4)	80 (25.9)	82 (33.3)	231 (54.2)	155 (55.2)	125 (64.8)	111 (59.7)	281 (51.6)	**1,074 (46.8)**
Myalgia	28 (26.2)	126 (40.8)	165 (67.1)	339 (79.6)	252 (89.7)	180 (93.3)	168 (90.3)	497 (91.2)	**1,755 (76.5)**
Arthralgia	17 (15.9)	115 (37.2)	159 (64.6)	338 (79.3)	249 (88.6)	175 (90.7)	172 (92.5)	501 (91.9)	**1,726 (75.3)**
Red or swollen joints or arthritis	20 (18.7)	51 (16.5)	62 (25.2)	143 (33.6)	121 (43.1)	105 (54.4)	97 (52.2)	325 (59.6)	**924 (40.3)**
**Total**	**107 (100.0)**	**309 (100.0)**	**246 (100.0)**	**426 (100.0)**	**281 (100.0)**	**193 (100.0)**	**186 (100.0)**	**545 (100.0)**	**2,293 (100.0)**

* Fever defined as either fever during the visit or in the past 7 days.

#### Zika Patient Characteristics and Symptoms

Among 1,918 confirmed or probable Zika cases, 1,612 (84.0%) were reported to SEDSS in 2016 (Figure 1) (Supplementary Table 2, https://stacks.cdc.gov/view/cdc/153721). Most participants (1,467 [76.5%]) visited SEDSS sites within 3 days of symptom onset (Table 3). A larger proportion of participants with Zika were female (57.7%) versus participants with dengue (46.6%) (p<0.001), and approximately half of visits by women with ZIKV infection who enrolled in SEDSS (544 [49.1%]) were of childbearing age; 77 (14.2%) were pregnant. The plurality of participants with laboratory evidence of ZIKV infection were aged ≥50 years (456 [23.8%]), followed by those aged 20–29 years (370 [19.3%]), 10–19 years (337 [17.6%]), and 30–39 years (261 [13.6%]). Most participants (1,770 [92.3%]) were discharged after the initial visit, whereas 117 (6.1%) were either admitted or transferred; one participant died. The most common comorbidities were hypertension (374 [19.5%]), asthma (341 [17.8%]), and diabetes (224 [11.7%]). Before laboratory testing, 82 (4.3%) participants received a clinical diagnosis of Zika, 131 (6.8%) with a respiratory infection, and 1,231 (64.2%) with an unspecified infection. A total of 152 (7.9%) participants with ZIKV infection were coinfected with respiratory viruses, 82 of whom had IAV or IBV.

In addition to fever and chills, the most reported common symptoms among the 1,918 participants with laboratory evidence of ZIKV infection were headache (1,493 [77.8%]), arthralgia (1,451 [75.7%]), and myalgia (1,405 [73.3%]). A total of 1,411 (73.6%) participants reported respiratory illness (e.g., nasal discharge, sore throat, and cough), and 1,089 (56.8%) reported gastrointestinal symptoms. The proportion of participants reporting symptoms such as myalgia, arthralgia, and headache consistently increased with age (p≤0.002) (Table 6).

**TABLE 6:** Number and percentage of cases of confirmed or probable Zika virus infection and clinical characteristics, by age — Sentinel Enhanced Dengue Surveillance System, Puerto Rico, 2012–2022

Symptom	<1 yr	1–4 yrs	5–9 yrs	10–19 yrs	20–29 yrs	30–39 yrs	40–49 yrs	≥50 yrs	Total
	No. (%)	No. (%)	No. (%)	No. (%)	No. (%)	No. (%)	No. (%)	No. (%)	No. (%)
Fever*	31 (96.9)	99 (99.0)	153 (99.4)	319 (94.7)	298 (80.5)	195 (74.7)	184 (88.5)	395 (86.6)	**1,674 (87.3)**
Chills	12 (37.5)	52 (52.0)	91 (59.1)	247 (73.3)	243 (65.7)	194 (74.3)	166 (79.8)	350 (76.8)	**1,355 (70.6)**
Rash	23 (71.9)	66 (66.0)	105 (68.2)	239 (70.9)	301 (81.4)	215 (82.4)	165 (79.3)	312 (68.4)	**1,426 (74.3)**
Headache	3 (9.4)	34 (34.0)	107 (69.5)	296 (87.8)	305 (82.4)	219 (83.9)	176 (84.6)	353 (77.4)	**1,493 (77.8)**
Eye pain	3 (9.4)	20 (20.0)	45 (29.2)	220 (65.3)	225 (60.8)	176 (67.4)	140 (67.3)	275 (60.3)	**1,104 (57.6)**
Myalgia	1 (3.1)	22 (22.0)	51 (33.1)	229 (68.0)	303 (81.9)	221 (84.7)	195 (93.8)	383 (84.0)	**1,405 (73.3)**
Arthralgia	1 (3.1)	17 (17.0)	61 (39.6)	231 (68.5)	311 (84.1)	227 (87.0)	196 (94.2)	407 (89.3)	**1,451 (75.7)**
Calf pain	1 (3.1)	11 (11.0)	34 (22.1)	129 (38.3)	172 (46.5)	144 (55.2)	151 (72.6)	282 (61.8)	**924 (48.2)**
Red or swollen joints or arthritis	0 (—)	6 (6.0)	17 (11.0)	59 (17.5)	137 (37.0)	123 (47.1)	108 (51.9)	230 (50.4)	**680 (35.5)**
Mucosal bleeding	2 (6.2)	17 (17.0)	22 (14.3)	40 (11.9)	68 (18.4)	50 (19.2)	35 (16.8)	60 (13.2)	**294 (15.3)**
Respiratory	25 (78.1)	77 (77.0)	118 (76.6)	264 (78.3)	268 (72.4)	169 (64.8)	157 (75.5)	333 (73.0)	**1,411 (73.6)**
Nasal discharge	17 (53.1)	64 (64.0)	74 (48.1)	158 (46.9)	123 (33.2)	89 (34.1)	80 (38.5)	186 (40.8)	**791 (41.2)**
Sore throat	5 (15.6)	32 (32.0)	71 (46.1)	203 (60.2)	210 (56.8)	120 (46.0)	116 (55.8)	240 (52.6)	**997 (52.0)**
Cough	20 (62.5)	57 (57.0)	84 (54.5)	148 (43.9)	153 (41.4)	93 (35.6)	98 (47.1)	225 (49.3)	**878 (45.8)**
Gastrointestinal illness	18 (56.2)	42 (42.0)	64 (41.6)	177 (52.5)	216 (58.4)	147 (56.3)	140 (67.3)	285 (62.5)	**1,089 (56.8)**
Nausea or vomiting	9 (28.1)	31 (31.0)	47 (30.5)	143 (42.4)	176 (47.6)	115 (44.1)	114 (54.8)	232 (50.9)	**867 (45.2)**
Diarrhea	13 (40.6)	22 (22.0)	34 (22.1)	94 (27.9)	124 (33.5)	88 (33.7)	89 (42.8)	167 (36.6)	**631 (32.9)**
**Total**	**32 (100.0)**	**100 (100.0)**	**154 (100.0)**	**337 (100.0)**	**370 (100.0)**	**261 (100.0)**	**208 (100.0)**	**456 (100.0)**	**1,918 (100.0)**

* Fever defined as either fever during the visit or in the past 7 days.

#### Respiratory Virus Patient Characteristics

Most confirmed respiratory virus cases were attributed to IAV (3,756), followed by SARS-CoV-2 (1,586), HAdV (1,550), RSV (1,489), IBV (1,430), HPIV-3 (993), HMPV (862), and HPIV-1 (407) (Table 7) (Figure 2) (Supplementary Table 3, https://stacks.cdc.gov/view/cdc/153721). A total of 1,842 participants with AFIs were tested for any of the HCoVs and 46 (2.5%) were infected, the majority of these cases were reported in 2012 and 2013 (41 [89.1%]) (Supplementary Figure 5, https://stacks.cdc.gov/view/cdc/153721). Among 11,922 participants testing positive for any of the respiratory viruses examined herein, 5,919 (49.7%) received a diagnosis of any respiratory virus before laboratory testing, whereas the remaining participants had AFI diagnosed that was attributed to other causes. The age distribution varied by virus identified. A plurality of cases with confirmed IAV or IBV infections were among participants aged 10–19 years (1,036 [20.0%]) followed by those aged 5–9 years (960 [18.5%]). Conversely, detection of RSV, HAdV, HPIV-1, HPIV-3, and HMPV was most frequent among those aged 1–4 years (758 [50.9%], 729 [47.0%], 222 [54.5%], 483 [48.6%], and 333 [38.6%]), respectively). RSV was the most frequent virus among infants aged <1 year (289 [19.4%]), whereas SARS-CoV-2 was most frequent among adults aged ≥50 years (36.4%). Most participants were discharged after triage; however, 367 (24.6%) of all participants with confirmed RSV infection were admitted or transferred. Nineteen participants with acute respiratory virus infections died; 12 were positive for SARS-CoV-2. The most common comorbidity across all participants with respiratory virus infections was asthma.

**TABLE 7:** Number and percentage of cases of respiratory viral illness (caused by influenza virus, respiratory syncytial virus, adenovirus, human parainfluenza virus, human metapneumovirus, human coronaviruses, and SARS-CoV-2) among all participants assessed and tested, by selected characteristics — Puerto Rico, Sentinel Enhanced Dengue Surveillance System, 2012–2022

Characteristic	Influenza A	Influenza B	Respiratory syncytial virus*	Adenovirus	Human parainfluenza virus 1	Human parainfluenza virus 3	Human metapneumovirus	Human coronavirus^†^	SARS-CoV-2
	No. (%)	No. (%)	No. (%)	No. (%)	No. (%)	No. (%)	No. (%)	No. (%)	No. (%)
**Emergency department physician diagnosis**									
Dengue	57 (1.5)	59 (4.1)	5 (0.3)	19 (1.2)	5 (1.2)	9 (0.9)	7 (0.8)	3 (6.5)	0 (—)
Chikungunya	7 (0.2)	2 (0.1)	0 (—)	2 (0.1)	0 (—)	3 (0.3)	1 (0.1)	0 (—)	0 (—)
Zika	2 (0.1)	2 (0.1)	2 (0.1)	1 (0.1)	1 (0.2)	1 (0.1)	2 (0.2)	0 (—)	0 (—)
Influenza	1,913 (50.9)	817 (57.1)	90 (6.0)	100 (6.5)	21 (7.4)	40 (5.1)	68 (7.9)	0 (—)	26 (1.6)
Leptospirosis	8 (0.2)	2 (0.1)	2 (0.1)	3 (0.2)	1 (0.2)	0 (—)	2 (0.2)	0 (—)	1 (0.1)
Respiratory infections^§^	657 (17.5)	248 (17.3)	679 (45.6)	373 (24.1)	65 (23.0)	266 (34.1)	372 (43.2)	14 (30.4)	460 (29.0)
Infection not otherwise specified^¶^	818 (21.8)	336 (23.5)	477 (32.0)	951 (61.4)	139 (49.3)	351 (45.0)	287 (33.3)	21 (45.7)	190 (12.0)
**Days post onset**									
0–3	3,396 (90.4)	1,226 (85.7)	1,188 (79.8)	1,284 (82.8)	357 (87.7)	825 (83.1)	696 (80.7)	35 (76.1)	1,161 (73.2)
4–7	342 (9.1)	198 (13.8)	282 (18.9)	249 (16.1)	48 (11.8)	159 (16.0)	155 (18.0)	11 (23.9)	308 (19.4)
>7	18 (0.5)	6 (0.4)	15 (1.0)	14 (0.9)	2 (0.5)	9 (0.9)	11 (1.3)	0 (—)	117 (7.4)
Missing	0 (—)	0 (—)	4 (0.3)	3 (0.2)	0 (—)	0 (—)	0 (—)	0 (—)	0 (—)
**Sex**									
Male	1,819 (48.4)	735 (51.4)	752 (50.5)	832 (53.7)	222 (54.5)	483 (48.6)	438 (50.8)	25 (54.3)	722 (45.5)
Female	1,937 (51.6)	695 (48.6)	737 (49.5)	718 (46.3)	185 (45.5)	510 (51.4)	424 (49.2)	21 (45.7)	864 (54.5)
**Age, yrs**									
<1	152 (4.0)	42 (2.9)	289 (19.4)	154 (9.9)	39 (9.6)	181 (18.2)	97 (11.3)	6 (13.0)	47 (3.0)
1–4	618 (16.5)	197 (13.8)	758 (50.9)	729 (47.0)	222 (54.5)	464 (46.7)	333 (38.6)	12 (26.1)	102 (6.4)
5–9	591 (15.7)	369 (25.8)	131 (8.8)	326 (21.0)	71 (17.4)	83 (8.4)	138 (16.0)	6 (13.0)	71 (4.5)
10–19	689 (18.3)	347 (24.3)	86 (5.8)	150 (9.7)	26 (6.4)	51 (5.1)	57 (6.6)	6 (13.0)	144 (9.1)
20–29	513 (13.7)	131 (9.2)	49 (3.3)	73 (4.7)	16 (3.9)	37 (3.7)	56 (6.5)	6 (13.0)	213 (13.4)
30–39	375 (10.0)	100 (7.0)	33 (2.2)	48 (3.1)	9 (2.2)	38 (3.8)	34 (3.9)	3 (6.5)	223 (14.1)
40–49	280 (7.5)	72 (5.0)	36 (2.4)	26 (1.7)	9 (2.2)	31 (3.1)	33 (3.8)	1 (2.2)	209 (13.2)
≥50	538 (14.3)	172 (12.0)	107 (7.2)	44 (2.8)	15 (3.7)	108 (10.9)	114 (13.2)	6 (13.0)	577 (36.4)
**Clinical disposition**									
Ambulatory	3,386 (90.1)	1,279 (89.4)	1,111 (74.6)	1,270 (81.9)	355 (87.2)	863 (86.9)	712 (82.6)	35 (76.1)	1,354 (85.4)
Admitted or transferred	342 (9.1)	142 (9.9)	367 (24.6)	277 (17.9)	51 (12.5)	125 (12.6)	142 (16.5)	10 (21.7)	205 (12.9)
Left against medical advice	11 (0.3)	4 (0.3)	3 (0.2)	1 (0.1)	0 (—)	3 (0.3)	5 (0.6)	1 (2.2)	2 (0.1)
Deceased	4 (0.1)	0 (—)	1 (0.1)	0 (—)	0 (—)	1 (0.1)	1 (0.1)	0 (—)	12 (0.8)
Unknown	13 (0.3)	5 (0.3)	7 (0.5)	2 (0.1)	1 (0.2)	1 (0.1)	2 (0.2)	0 (—)	13 (0.8)
**Co-existing medical condition**									
Diabetes	269 (7.2)	98 (6.9)	49 (3.3)	28 (1.8)	15 (3.7)	61 (6.2)	68 (7.9)	5 (10.9)	220 (13.9)
Chronic pulmonary disease	720 (19.2)	302 (21.1)	264 (17.7)	263 (17.0)	85 (20.9)	152 (15.3)	192 (22.3)	7 (15.2)	345 (21.8)
Hypertension	434 (11.6)	133 (9.3)	85 (5.7)	46 (3.0)	12 (2.9)	97 (9.8)	90 (10.4)	5 (10.9)	389 (24.5)
Chronic kidney disease	19 (0.5)	9 (0.6)	7 (0.5)	7 (0.5)	1 (0.2)	8 (0.8)	10 (1.2)	2 (4.3)	36 (2.3)
Immunodeficiency	17 (0.5)	8 (0.6)	4 (0.3)	2 (0.1)	2 (0.5)	2 (0.2)	9 (1.0)	1 (2.2)	12 (0.8)
Sickle cell disease	15 (0.4)	5 (0.3)	5 (0.3)	5 (0.3)	0 (—)	0 (—)	3 (0.3)	0 (—)	4 (0.3)
Obesity**	712 (24.4)	185 (17.3)	130 (11.1)	103 (9.9)	27 (9.6)	110 (14.1)	117 (18.0)	5 (29.4)	550 (38.3)
**Pregnant^††^**									
Yes	66 (8.9)	19 (9.5)	3 (3.3)	4 (4.1)	4 (17.4)	7 (8.8)	5 (6.5)	1 (25.0)	9 (2.5)
No	642 (86.3)	180 (89.5)	80 (88.9)	93 (94.9)	18 (78.3)	70 (87.5)	72 (93.5)	2 (50.0)	347 (95.6)
Unknown	36 (4.8)	2 (1.0)	7 (7.8)	1 (1.0)	1 (4.3)	3 (3.7)	0 (—)	1 (25.0)	7 (1.9)
Visits from women aged 15–44, no.	744	201	90	98	23	80	77	4	363
**Coinfections**									
Arboviral co-infection	77 (2.1)	74 (5.2)	28 (1.9)	55 (3.5)	9 (2.2)	33 (3.3)	40 (4.6)	6 (13.0)	2 (0.1)
Respiratory virus co-infection	78 (2.1)	25 (1.7)	71 (4.8)	106 (6.8)	19 (4.7)	40 (4.0)	43 (5.0)	5 (10.9)	9 (0.6)
**Total**	**3,756 (100.0)**	**1,430 (100.0)**	**1,489 (100.0)**	**1,550 (100.0)**	**407 (100.0)**	**993 (100.0)**	**862 (100.0)**	**46 (100.0)**	**1,586 (100.0)**

* Respiratory syncytial virus includes subtypes A and B.

^†^ Human coronavirus includes types 229E, OC43, NL63, and HKU1.

^§^ Respiratory infections include pneumonia, upper respiratory tract infections, bronchitis, bronchiolitis, respiratory syncytial virus, and COVID-19.

^¶^ Infections not otherwise specified include urinary tract infections, gastroenteritis, pharyngitis, fever unspecified, viral syndrome, tonsillitis, otitis media, sinusitis, impetigo, cellulitis, infected wound, lymphadenopathy cervical, herpangina, febrile seizure, meningitis, sepsis, bacteremia, encephalitis, pancreatitis, myositis, conjunctivitis, myocarditis, Guillain-Barré syndrome, mononucleosis, and pyelonephritis.

** Among persons with available body mass index. Influenza A (n = 2,917), influenza B (n = 1,430), respiratory syncytial virus (n = 1,167), adenovirus (n = 1,037), human parainfluenza virus 1 (n = 282), human parainfluenza virus 3 (n = 780), human metapneumovirus (n = 651), human coronavirus (n = 17), and SARS-CoV-2 (n = 1,437).

^††^ Denominators for these analyses include visits from women of childbearing age defined as any female aged 15–44 years.

## Discussion

Initially designed for enhanced dengue surveillance, SEDSS has evolved into a valuable and nimble AFI surveillance system, providing insights into the early epidemiology of various emerging pathogens, including CHIKV, ZIKV, and SARS-CoV-2, while also monitoring trends in influenza and other respiratory viruses during the surveillance period. The system’s flexibility allowed for the rapid setup of studies to understand new viruses and support the development of public health guidelines, exemplified by the successful implementation of a study to evaluate the duration of ZIKV RNA in body fluids (*26*) as well as the evaluation of rapid, point-of-care testing for SARS-CoV-2 detection and an IgM assay to distinguish between DENV and ZIKV infections (*27*,*28*). Other studies leveraged SEDSS to examine epidemiologic, clinical, and spatiotemporal patterns of Zika, chikungunya, leptospirosis, and COVID-19 in Puerto Rico (*29*–*32*).

In contrast to the limitations of passive surveillance (e.g., underreporting and delayed case identification), SEDSS facilitated real-time detection and collection of detailed information about participants affected by arboviruses and respiratory viruses. This adaptability highlights the value of SEDSS as a surveillance tool for novel infectious threats, addressing the limitations associated with relying solely on passive surveillance. The expansion of SEDSS’s scope allowed for the documentation of temporal variations in both endemic arboviruses and respiratory viruses, providing a more comprehensive understanding of disease dynamics. Furthermore, this analysis assessed disease severity, including the proportion of participants with warning signs and those requiring hospitalization, aiding in clinical care evaluation. These data are useful in evaluating the health care system’s response to these diseases, offering insights that can guide future interventions, research gaps, and clinical care guidelines.

Among the three arboviruses, the chikungunya epidemic in 2014 recorded the highest number of participants with confirmed disease reported through SEDSS (2,293 cases). This observation aligns with observations from other regions in the Western Hemisphere that similarly experienced intense periods of CHIKV transmission among immunologically naïve populations in 2014 (*33*). Chikungunya typically has a higher symptomatic attack rate than Zika, resulting in a larger number of reported cases compared with other arboviruses, which might contribute to observed differences in case numbers between chikungunya and Zika (*34*,*35*). ZIKV was the second most reported arbovirus in SEDSS, with 1,918 cases reported. The first Zika case reported through SEDSS in January 2016 followed the first cases reported islandwide in December 2015. ZIKV transmission slowed after the first quarter of 2017, with the last confirmed case reported in April 2018 and low numbers of probable cases reported through 2019. The presence of persistent ZIKV IgM antibodies in these cases might not necessarily indicate recent infection because ZIKV IgM antibodies can persist for an extended period after the acute phase of infection, complicating the interpretation of serologic results (*36*). Cross-reactivity with DENV antibodies further challenges precise diagnosis. Since 2019, patients among all locally acquired Zika cases in U.S. territories had Zika diagnosed by antibody testing (probable cases), which cannot distinguish recent from past infections. Although the total number of dengue cases reported through SEDSS was lower, the most recent dengue outbreak persisted for >2 years. The lower transmission intensity of dengue compared with chikungunya and Zika likely reflects a certain degree of population immunity from previous DENV infections during the outbreak periods. No confirmed dengue cases were reported through SEDSS in 2017 and 2018, the years immediately following the Zika epidemic. This observation comports with a stochastic compartmental model trained on surveillance data from Colombia and Brazil (accounting for combinations of enhancement or cross-protection) that supports cross-protection against dengue for 2.2–3.5 years after a Zika outbreak (*37*) and precedes dengue resurgence. Insights from studies in Thailand and Nicaragua suggest that contrary to a 2-year waning period, cross-reactive binding antibodies linked to protection or enhancement reach a stable setpoint approximately 8 months after primary infection and persist for many years afterward (*38*,*39*). Although cross-protection provides a partial explanation, other factors (e.g., public health interventions such as mosquito control programs, educational campaigns, and vector surveillance) could have been intensified in response to the Zika outbreak, potentially contributing to the decrease in DENV transmission.

Most participants affected by any of the three arboviruses were aged 5–50 years, but disproportionately more participants aged ≥50 years had Zika or chikungunya compared with the age distribution of the overall SEDSS population. Relatively few cases among infants aged <1 year were reported through SEDSS with any of the three arboviruses, but more cases among infants were reported with laboratory evidence of chikungunya infection (4.7% of the overall population) compared with dengue and Zika (2.0% and 1.7% of the overall population, respectively). This observation might reflect the possible higher morbidity including cutaneous alterations and arthralgia or arthritis associated with chikungunya infection in infancy (*40*,*41*) compared with the low relative morbidity associated with primary dengue or Zika infections (*42*). Twenty-seven percent of participants with dengue progressed to severe dengue. However, only 10.1% of children aged 1–4 years had severe dengue, which could reflect lower likelihood of experiencing antibody-dependent enhancement because of infection from other serotypes during previous outbreaks (*43*,*44*).

Fewer pregnant women were reported through SEDSS with evidence of dengue infection (4.6%) compared with Zika (14.2%) or chikungunya (14.0%). The higher proportion of pregnant women reported through SEDSS with evidence of confirmed or probable ZIKV infection could be because of increased health care-seeking behavior by pregnant women during the Zika epidemic (*45*).

The highest proportion of participants testing positive for any arbovirus was observed among residents in southeast Puerto Rico, particularly in Guayama. Urbanization and population density are associated with *Aedes aegypti* abundance, driven by favorable breeding conditions, higher larval development rates, and adult survival times, especially in rapidly urbanizing areas with unplanned settlements (*46*,*47*). Previous research has confirmed high mosquito populations in southern Puerto Rico (*48*,*49*). Abandoned and inhabited houses in lower socioeconomic neighborhoods in southern Puerto Rico contribute to *Ae. aegypti* pupal productivity, emphasizing the need for targeted vector control strategies in these neighborhoods (*49*).

These findings highlight challenges and gaps in arboviral disease management with a focus on dengue. First, many participants with confirmed dengue, Zika, and chikungunya were initially misdiagnosed with other diseases (e.g., respiratory illnesses) by ED physicians. Obtaining an accurate diagnosis based on clinical presentation alone is challenging, as demonstrated by systemic reviews of the accuracy of clinical definitions of dengue (*50*,*51*). Certain symptoms (e.g., headache) increased with age for dengue and Zika cases, and these findings were consistent with another SEDSS study that focused on children; however young children might be less likely to vocalize these symptoms (*12*). Another study using SEDSS data also reported overlapping symptoms between dengue and chikungunya, but patients receiving a diagnosis of chikungunya were more likely to report arthritis, skin rash, and irritability, and less likely to have signs of poor circulation, diarrhea, headache, and cough (*52*). Age-related symptom variations and frequent coinfections with respiratory viruses (e.g., IAV, IBV, and HAdV) highlight the complexity of arboviral infections and the necessity for precise diagnostic approaches that consider multiple pathogens for timely and accurate diagnoses (*8*,*12*).

Second, 37% of all participants with a dengue diagnosis who had warning signs and 84% of those with severe dengue diagnosed were hospitalized. The most recent guidelines recommend hospitalization for suspected patients with dengue and having warning signs or severe dengue (*53*). Among participants with warning signs or severe dengue who were not hospitalized, 17.6% received a correct initial dengue diagnosis. Taken together, these findings highlight the need for ongoing training for physicians on dengue clinical management guidelines and the need for more rapid detection of dengue. Moreover, these findings also underscore the potential strain on the health care system when a substantial proportion of patients necessitate hospitalization. SEDSS plays a pivotal role in enabling precise disease diagnosis and addressing the challenge of misclassification (*29*,*54*); however, results are still not prompt enough to guide clinical decision making at the bedside. Implementation of rapid diagnostic testing for dengue, currently unavailable in the United States or its territories, could expedite the identification of patients requiring hospitalization and address gaps in clinical care.

SEDSS continues to be used to monitor endemic arboviruses in Puerto Rico and acts as a sentinel for emerging pathogens which initially might be missed by clinical diagnosis. Approximately 545 recognized arboviruses exist worldwide, although estimates suggest this figure might represent less than 1% of all arboviruses because most are zoonotic infections among hosts other than humans (*55*–*57*). Among the recognized arboviruses, approximately 150 have been documented to cause human disease (*55*). This observation highlights the importance of robust surveillance systems like SEDSS, which are essential for timely identification and monitoring of emerging infectious threats that might otherwise go undetected.

Distinct seasonality in the circulation of respiratory viruses was observed, consistent with findings documented in the literature on virus-virus interactions and their effect on disease dynamics (*58*–*60*). A related study using SEDSS found distinct seasonal patterns in Puerto Rico, with respiratory viruses peaking in fall and winter, while arboviruses (e.g., dengue) peaked in summer and early fall, emphasizing the importance of recognizing these seasonal trends for accurate diagnosis and public health interventions in pediatric patients with AFI (*10*). Viral interference, a phenomenon highlighted in previous research, plays a role in shaping the observed patterns (*61*,*62*). At the population level, viral interference might be induced when an outbreak caused by one virus hastens or delays an epidemic caused by another virus (*58*,*60*). These results indicate that the seasonality of IAV typically precedes that of IBV each year, consistent with documented interactions between influenza types and subtypes that might involve cross-immunity mechanisms (*63*,*64*). Another study using SEDSS data analyzed influenza trends in Puerto Rico during 2012–2018, illustrated that influenza seasonality in the region differed from that in the continental United States, with irregular and asynchronous epidemics initially but increased synchrony in recent years (*54*). The study suggests that factors beyond climate, such as travel and viral introductions, might contribute to the observed changes in influenza dynamics in Puerto Rico.

In addition, the strong seasonality observed for RSV reflects patterns observed in various regions, with increasing RSV epidemics over the years, particularly among children (*65*). The findings from this report revealed a high proportion of acute respiratory viral cases among children aged <5 years, including RSV, HPIV-3, HPIV-1, HAdV, and HMPV. Children, particularly those aged <5 years, have increased susceptibility to viral infections compared with adults, possibly because of higher contact frequencies with peers in school and child care settings, naïve immune systems, limited previous exposure, and a greater likelihood of medical care-seeking by parents (*60*,*66*).

From March 2020 to April 2021, nearly all respiratory viral infections identified were SARS-CoV-2, demonstrating its high transmissibility compared with other respiratory pathogens (*67*,*68*). The COVID-19 pandemic considerably altered RSV seasonality, with atypical transmission patterns observed in mid-2021 and during September–November 2022 (*69*). These trends in Puerto Rico align with those in the continental United States (*69*). Findings from this report align with recent research on the impact of the emergence of SARS-CoV-2, which led to a substantial reduction in the circulation of other acute respiratory viruses (*70*). Previous analyses conducted using data from SEDSS during the 2019–2020 respiratory virus season revealed a considerable reduction in the test positivity of IAV, IBV, RSV, HAdV, and other respiratory viruses following the implementation of COVID-19 stay-at-home orders (*71*). The reductions in test positivity persisted even after the initial impact of COVID-19 mitigation measures, indicating a sustained effect on the transmission of respiratory infections. This reduction is attributed to a combination of changes in human interactions, nonpharmaceutical interventions, reduced testing of seasonal respiratory viruses, and potential viral interferences (*70*).

The results presented in this study should be interpreted in the context of external factors that could influence disease dynamics. Puerto Rico has been increasingly vulnerable to climate change, characterized by more frequent and severe hurricanes. Extreme weather events, such as hurricanes, can disrupt health care infrastructure and disease surveillance efforts. The erosion of infrastructure during severe storms might have heightened the occurrence of certain diseases and led to population movements, potentially affecting population immunity. Severe storms might increase the number of mosquitoes by filling containers with rainwater, increasing the edge surface of groundwater habitats, and generating additional container aquatic habitats in destroyed or abandoned properties. This increase in aquatic habitats was observed after Hurricanes Irma and Maria in Caguas, where adult female *Ae. aegypti* doubled in numbers after the hurricanes, possibly because of an increase in the number of containers holding water, even in years with similar rainfall (*72*). The impact of these climatic disruptions, natural disasters, and population dynamics on disease patterns in Puerto Rico needs further investigation. In addition, climate change can alter the distribution and behavior of disease vectors, potentially affecting the geographic range of arboviral diseases (*73*).

## Limitations

The findings in this report are subject to at least six limitations. First, SEDSS was established at certain sentinel sites based on historically high dengue incidence. Therefore, these results might not be reflective of all of Puerto Rico. The results included in this report were derived principally from sentinel sites in southern Puerto Rico as well as the San Juan metro area; the geographic distribution of participant residences are skewed toward these regions. Second, the symptoms in the SEDSS clinic intake and follow-up forms were based on participant self-report and, in certain instances, were not sufficient to differentiate between dengue warning signs and signs of severe dengue. For example, SEDSS intake forms could capture self-reported mucosal bleeding (e.g., nose and gum bleeding, gastrointestinal bleeding, and excessive vaginal bleeding), but these self-reports could not clinically distinguish severe bleeding from the gastrointestinal tract (e.g., hematemesis and melena) or vagina (e.g., menorrhagia) as defined by the requirement for medical intervention, including intravenous fluid resuscitation or blood transfusion (*74*). Inpatient data, where available, were merged with the SEDSS data and enabled further dengue severity differentiation. Third, differentiating between dengue and Zika presented challenges after Zika emerged because probable dengue diagnosis relied on IgM tests for DENV with negative results for ZIKV and vice versa for probable Zika diagnosis because of cross-reactivity between dengue and Zika IgM antibodies in serologic tests. Fourth, SEDSS is a convenience sample, and recruited cases represent a fraction of total eligible patients (Supplementary Table 1, https://stacks.cdc.gov/view/cdc/153721). Fifth, the subjective nature of some self-reported symptoms (e.g., restlessness and abdominal pain) might have contributed to over-reporting of dengue warning signs, while health care provider-only warning signs (e.g., abdominal tenderness, liver enlargement, or substantial changes in hematocrit or platelet counts over two measurements) could have been missed. Subsequent iterations of intake and follow-up forms will address this issue in current and future years. Finally, SEDSS, while offering real-time and detailed information, presents challenges because of its labor-intensive nature and higher costs compared with passive surveillance methods, posing resource and infrastructure demands that might be challenging for implementation in various countries.

## Future Direction

In addition to contributing to research and public health efforts, SEDSS also provides a valuable system for piloting health care provider diagnostic support tools. The data in this report guide evidence-based strategies for preventing and controlling arboviral diseases and emerging pathogens with global relevance beyond Puerto Rico. Puerto Rico’s experience with febrile illness surveillance provides a model for similar applications in other areas where *Ae. aegypti* is prevalent. This is particularly relevant considering the potential of changes in climate and weather, which could lead to rising global temperatures and expand the geographic range of disease vectors (*75*). Leveraging the lessons from SEDSS, an opportunity to enhance health care system resilience by improving diagnostic capabilities, increasing surge capacity, and ensuring the stockpiling of essential medical supplies exists. SEDSS has the potential to integrate into global surveillance networks, strengthening international collaboration for early detection and response to emerging infectious diseases. Expansion of SEDSS to additional sentinel sites can supplement and enhance information provided through existing passive reporting mechanisms and improve the geographic representation of this active surveillance system.

SEDSS is uniquely positioned to provide a source population for future real-world effectiveness studies of novel vaccines for arboviral diseases. These vaccines might include vaccines like the live-attenuated, single-dose VLA1553 for chikungunya (*76*) and dengue antivirals (e.g., JNJ-1802) (*77*). Data analytics, modeling, and machine learning could be applied to SEDSS data to help identify disease trends, predict outbreaks, and optimize resource allocation for public health responses. SEDSS data might provide a quantitative measure of effectiveness for vector control strategies (e.g., novel insecticides and genetically modified mosquitoes) derived from observed case counts before and after intervention implementation. Prioritizing capacity building and training for local health care providers, researchers, and public health personnel is essential to enhance expertise in disease surveillance and management, ultimately improving responses to future outbreaks both in Puerto Rico and elsewhere.

## Conclusion

SEDSS enables in-depth patient tracking and follow-up to identify common and emerging arboviral and respiratory viruses causing AFI. SEDSS also facilitates understanding the temporal patterns of these viruses, potential interactions between them, and the populations most at risk for illness. In addition, SEDSS proved adaptable in detecting and collecting detailed information on participants affected by emerging arboviruses in Puerto Rico (e.g., CHIKV and ZIKV) and numerous respiratory viruses, including SARS-CoV-2. SEDSS serves as a versatile model for effective disease surveillance, offering valuable insights applicable beyond the context of Puerto Rico in the dynamic field of infectious disease monitoring.
